# Evidence-based guidelines for use of probiotics in preterm neonates

**DOI:** 10.1186/1741-7015-9-92

**Published:** 2011-08-02

**Authors:** Girish C Deshpande, Shripada C Rao, Anthony D Keil, Sanjay K Patole

**Affiliations:** 1 Department of Neonatal Paediatrics, Nepean Hospital Sydney, Sydney, Australia; 2 University of Sydney, Australia Sydney, Australia; 3 Department of Neonatal Paediatrics, KEM Hospital for Women, Perth, Australia; 4 Department of Neonatal Paediatrics, Princess Margaret Hospital for Children, Perth, Australia; 5 University of Western Australia, Perth, Australia; 6 PathWest Laboratory Medicine, WA, USA

## Abstract

**Background:**

Current evidence indicates that probiotic supplementation significantly reduces all-cause mortality and definite necrotising enterocolitis without significant adverse effects in preterm neonates. As the debate about the pros and cons of routine probiotic supplementation continues, many institutions are satisfied with the current evidence and wish to use probiotics routinely. Because of the lack of detail on many practical aspects of probiotic supplementation, clinician-friendly guidelines are urgently needed to optimise use of probiotics in preterm neonates.

**Aim:**

To develop evidence-based guidelines for probiotic supplementation in preterm neonates.

**Methods:**

To develop core guidelines on use of probiotics, including strain selection, dose and duration of supplementation, we primarily used the data from our recent updated systematic review of randomised controlled trials. For equally important issues including strain identification, monitoring for adverse effects, product format, storage and transport, and regulatory hurdles, a comprehensive literature search, covering the period 1966-2010 without restriction on the study design, was conducted, using the databases PubMed and EMBASE, and the proceedings of scientific conferences; these data were used in our updated systematic review.

**Results:**

In this review, we present guidelines, including level of evidence, for the practical aspects (for example, strain selection, dose, duration, clinical and laboratory surveillance) of probiotic supplementation, and for dealing with non-clinical but important issues (for example, regulatory requirements, product format). Evidence was inadequate in some areas, and these should be a target for further research.

**Conclusion:**

We hope that these evidence-based guidelines will help to optimise the use of probiotics in preterm neonates. Continued research is essential to provide answers to the current gaps in knowledge about probiotics.

## Background

Despite the advances in neonatal intensive care over past 20 years [1], the incidence of necrotising enterocolitis (NEC) in preterm neonates has not changed significantly. The mortality (approximately 20 to 25%) and morbidity related to definite (greater than stage II) NEC, including prolonged hospitalisation [2], survival with short-bowel syndrome [3,4] and long-term neurodevelopmental impairment (NDI) continues to be high, especially in preterm or extremely low birth weight (ELBW) (birth weight < 1000 g, gestation < 28 weeks) neonates needing surgery for this illness [5]. Mortality reaches nearly 100% in children with extensive and full-thickness necrosis of the gut [6,7].

Antenatal use of glucocorticoids, with postnatally, preferential feeding with fresh human milk, aggressive prevention and treatment of sepsis, and a cautious uniform approach to enteral feeds are the strategies available to prevent NEC [8]. Previous systematic reviews of randomised controlled trials (RCTs) showed that probiotic supplementation significantly reduces the risk of definite NEC, all-cause mortality and the time to reach full enteral feeds (~120 to 150 ml/kg/day of milk) in preterm neonates [9-11]. Based on these results, reports have indicated that routine probiotic supplementation is justified, except for ELBW neonates, given the lack of specific data on this high-risk cohort [12,13]. Our most recent updated systematic review and meta-analysis confirmed previous results, while improving their precision and reducing the likelihood of these being due to chance alone (Table 1) [14]. Moreover, trial sequential analysis (TSA) indicated that the results gave conclusive evidence of at least 30% reduction in the incidence of NEC. These conclusive results, along with those from observational studies on routine use of probiotics, their use in ELBW neonates [15,16], and their safety and possible benefits in terms of long-term NDI, justify a change in practice if safe and suitable probiotic products are available [17]. Some have supported our views [18], but others cite difficulties such as problems in pooling data in the presence of clinical heterogeneity, reproducibility of the results in different studies, role of breast milk, pitfalls of TSA, lack of availability of safe and effective products, development of antibiotic resistance, cross-contamination and long-term adverse effects (AEs) as reasons for opposing routine use of probiotics in preterm neonates [19-23]. We have previously addressed these concerns [24], and pointed out that probiotic research has completed a full circle, from basic science [25] and cohort studies [26], to conclusive meta-analysis [14], routine use [15], and long-term follow up [16,17]. Many level III neonatal units in Japan, Italy, Finland and Columbia have been using probiotics routinely for over a decade, and have not reported any significant AEs [15,27,28]. Based on the quality and totality of the evidence in the context of the related health burden and the lack of equally effective therapies, we believe that probiotics should be offered routinely to preterm neonates. Additionally, from the perspective of a preterm neonate or their family, there would need to be a good reason to ignore the evidence base for using probiotics to prevent NEC. Offering probiotics routinely, but still within a framework of research other than placebo-controlled trials, is the way forward to deal with the as yet unanswered questions [14,24]. As the debate about the pros and cons of routine probiotic supplementation continues, many institutions are satisfied with the current evidence and wish to use probiotics routinely. Because of the lack of detail on many practical aspects of probiotic supplementation, clinician-friendly guidelines are urgently needed to optimise use of probiotics in preterm neonates.

**Table 1:** Updated systematic review results (Pediatrics 2010)

Outcome	**RR**^**a**^**(95% CI)**	*P *value	**NNT**^**b **^**(95% CI**^**c **^**)**
NEC	0.35 (0.23 to 0.55)	0.00001	25 (17 to 34)
Mortality	0.42 (0.29 to 0.62)	0.00001	20 (14 to 34)
Sepsis	0.98 (0.81 to 1.18)	0.80	N/A^**d**^

^a^Relative risk.

^b^Confidence interval.

^c^Numbers needed to treat.

^d^Not available.

Because of the vast scope of the field, we aimed to conduct a comprehensive rather than a conventional systematic review in order to develop evidence-based guidelines for using probiotics in preterm neonates, and we indicate areas for further exploration of this new frontier.

## Methods

To develop the core guidelines for strain selection, age at start, dose and duration of the supplementation, we primarily used the data from RCTs of probiotics in preterm neonates from our recent updated systematic review [14].

For equally important issues such as strain identification, AEs, product format, storage and transport, regulatory issues, ethics and parent information, the relevant literature was searched in PubMed (1966 to October 2010) and EMBASE for the period 1980 to October 2010, and we also used the search engine Google.

PubMed was searched using the following terms: "Probiotics"[MeSH] AND "Culture Techniques"[MeSH]; "Probiotics"[MeSH] AND "Classification"[MeSH]; "Probiotics"[MeSH] AND "Bacterial Translocation"[MeSH]; "Probiotics"[MeSH] AND "Sepsis"[MeSH]; "Probiotics"[MeSH] AND "Informed Consent"[MeSH]; "Probiotics"[MeSH] AND "Legislation, Drug"[MeSH]; "Probiotics"[MeSH] AND ("Ethics"[MeSH] OR "Ethics Committees"[MeSH] OR "Ethics Committees, Clinical"[MeSH] OR "Codes of Ethics"[MeSH] OR "Ethics Committees, Research"[MeSH] OR "Ethics, Clinical"[MeSH] OR "Ethics, Professional"[MeSH] OR "Ethics, Medical"[MeSH] OR "Bioethics"[MeSH]); "Probiotics"[MeSH] AND Refrigeration"[MeSH] "Probiotics"[MeSH] AND "Quality Control"[MeSH]; "Probiotics"[MeSH] AND "Quality Assurance, Health Care"[MeSH].

EMBASE was searched using the following terms: probiotic.mp. or probiotic agent AND microbiological examination/or culture medium/or methodology/or culture methods.mp. or culture technique/or bacterium culture/; probiotic.mp. or probiotic agent AND antibiotic susceptibility.mp. or antibiotic sensitivity; probiotic.mp. or probiotic agent AND Sepsis; probiotic.mp. or probiotic agent AND bacterial translocation; probiotic.mp. or probiotic agent AND legislation.mp. or licence/or law/; probiotic.mp. or probiotic agent AND informed consent; probiotics.mp. or probiotic agent AND temperature/or drug storage/or drug packaging/or cold chain.mp. or drug stability/or freezing/; probiotic.mp. or probiotic agent quality assurance.mp. or quality control/.

The search covered studies in the neonatal, paediatric and adult populations, and also in animal studies and *in vitro *studies. Cross-references from the relevant studies were also searched. Specific references that were used to develop the guidelines are quoted in the main manuscript of the review. All other essential or related references are included in the appendices (see Additional file 1; see Additional file 2), which also include the results of the PubMed and EMBASE search strategies.

An attempt to search Google search engine using the aforementioned terms was abandoned, as it resulted in hits ranging from 838 to 1,690,000. PRISMA guidelines for reporting the systematic review were followed where applicable [29].

When establishing guidelines, it is preferable to grade the level of evidence (LOE) depending on the type and the quality of study. However, we found that there are no validated and universally accepted methods for assessing the quality of studies) especially for studies other than RCTs), or for grading the LOE [30-37]. Our core guidelines are based on the systematic review of RCTs of probiotic supplementation in preterm very low birth weight (VLBW) neonates. The quality of these trials was assessed by the method recommended by the Cochrane Neonatal Review Group and by Jadad scores, which are commonly used but have not been validated [38,39].

**Figure 1:**
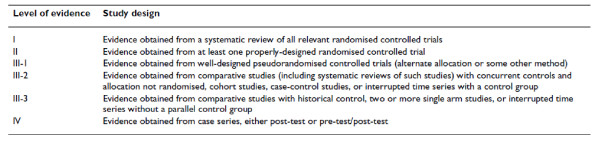
**Designations of levels of evidence**. Figure from *Merlin et al*. *BMC Medical Research Methodology *2009: 34, doi:10.1186/1471-2288-9-34.

The development and reporting of crucial aspects of probiotics (for example, selection, manufacturing, transport, storage, quality control (QC), and regulation) has not necessarily followed the model of evidence-based medicine, making it difficult to apply the principles of LOE to every aspect of this intervention. It is also difficult to apply the conventional concept of study design and LOE for bench research to practical issues such as stability and taxonomy confirmation. We therefore adopted a simple method of grading the LOE, based on a pyramid of evidence hierarchy, with systematic reviews of RCTs being at the top (best evidence) and a case series being the bottom [33] (Figure 1). We believe that this simple system for grading the LOE along with the judgement of the readers will be adequate to permit appropriate interpretation of the various aspects of the guidelines.

## Results

### Selection of strains

Bifidobacteria and lactobacilli are the species of choice in probiotics, given the evolution of the gut flora in preterm neonates [40-42]. However, lactobacilli are a minor component of the intestinal microbiota. In terms of the rationale for species and strain selection, it is important to note that there are many different mechanisms producing the benefits of probiotics and there are also strain-specific effects. Bifidobacteria are the dominant strains in infancy, and the combination of lactobacilli and bifidobacteria is known to promote the growth of indigenous lactic-acid bacteria (bifidogenic effect) by formation of short-chain fatty acids as a product of the fermentation process [41,43,44].

Strains isolated from humans are preferable because of their natural occurrence, long-term record of safety in infants, and adaptability to both mucosal and dairy ecosystems [45,46]. Researchers have generally selected strains belonging to bacterial species naturally present in the intestinal flora of the species to be targeted (in this case, humans), under the assumption that these bacteria have a better chance of out-competing resident bacteria and of establishing themselves at a numerically significant level in their new host [45]. However, because humans have shared such strains with other mammals for millions of years, other researchers believe that their origin is difficult to trace as they are present everywhere: in human beings, animals, soil, food and water. Thus, bovine strains may also be used if they have a good record of safety and efficacy. It is the specificity of the action rather than the source of the microorganism that is important. The clinical significance of the origin of strains may be evaluated in future trials [45,47-51]. The probiotic strains that have been used in various RCTs in preterm neonates are summarised in Table 2[52-62].

**Table 2:** Probiotic protocols from trials included in the updated meta-analysis

Study	Probiotic agent/s	Dose and duration
Kitajima 1997 [52]	*Bifidobacterium breve*	0.5 × 10^9 ^cfu^a ^once daily from first feed for 28 days.

Dani 2002 [53]	*Lactobacillus rhamnosus *GG	6 × 10^9 ^cfu once daily from first feed until discharge

Costalos 2003 [54]	*Saccharomyces boulardii*	10^9^/kg twice daily from first feed for 30 days

Bin Nun 2005 [55]	*Bifidobacterium infantis*, *Streptococcus thermophilus*, *Bifidobacterium bifidus*	0.35 × 10^9 ^cfu *B. infantis*, 0.35 × 10^9 ^cfu *S. thermophilus *and 0.35 × 10^9 ^cfu *B. bifidus *once daily from first feed to 36 weeks corrected age

Lin 2005 [56]	*Lactobacillus acidophilus*, *B. infantis*	1004356 *L. acidophilus *and 1015697 *B. infantis *organisms twice daily from day 7 until discharge

Manzoni 2006 [57]	*Lactobacillus casei*	6 × 10^9 ^cfu once daily from 3 days to 6 weeks of age, or discharge from NICU^b^

Mohan 2006 [28]	*Bifidobacterium lactis*	1.6 × 10^9 ^cfu once daily from day 1 to day 3; 4.8 × 10^9 ^cfu once daily from day 4 to day 21

Stratiki 2007 [59]	*B. lactis*	Preterm formula: 1 × 10^7 ^cfu/g started within 48 hours to 30 days

Lin 2008[60]	*B. bifidus*, *L. acidophilus*	2 × 10^9 ^cfu daily for 6 weeks

Samanta 2009[61]	*B. bifidus*, *B. lactis*, *B. infantis*, *L.s acidophilus*	2.5 × 10^9 ^cfu daily until discharge

Rouge 2009 [62]	*Bifidobacterium longum*, *Lactobacillus *GG	1 × 10^8 ^cfu daily until discharge

^a^Colony-forming units.

^b^Neonatal intensive-care unit.

It is important to note that the probiotic effects are strain-specific, and cannot be extrapolated to other strains. The variability of the strains and protocols in the trials included in our meta-analysis indicates that the concept of strain-specific effects of probiotics may not be relevant to prevention of NEC by probiotics. Because of the various complex pathways involved in the pathogenesis of NEC, different strains may benefit by different pathways [63]. The benefits of different probiotics in infective diarrhea indicate that although many probiotic effects are strain-specific, others may be similar for very different probiotic organisms [64]. The variation in the immunomodulatory effects between species is generally larger than that between the strains of the same species [65]. The rates of gut colonisation by a probiotic strain also differ according to the age of the host [52,66,67].

Evidence indicates that the functionality of a multistrain or multispecies probiotic could be more effective and more consistent than that of a monostrain probiotic [46,68,69]. Researchers have also investigated the adequacy of combinations of strains [70]. Colonisation of an ecosystem providing a niche for more than 400 species in combination with individually determined host factors is anticipated to be more successful with multistrain rather than monostrain probiotic preparations [46,50,71,72]. The results of one review indicated that multistrain probiotics showed greater efficacy than single strains, including single strains that were components of the mixtures themselves. It was unclear whether this was due to synergistic interactions between strains or to the higher probiotic dose used in some studies [73]. Based on the complexity of normal gut flora and of NEC pathogenesis, and the multiple beneficial mechanisms of probiotic strains, multistrain probiotics may be more effective than single-strain probiotics [63,74]. However, the report of a consensus meeting of experts states that a combination of probiotic strains in a product does not necessarily add to the benefits of each strain [75]. A high number of different strains is not, in itself, indicative of greater efficacy than a lower number of strains [75]. Clinical trials are needed to address the benefits of single-versus multistrain probiotic products in preterm neonates.

Our systematic review of RCTs indicates that the trials reporting a significant decline in NEC used multistrain products [55,56,60], whereas those reporting a lesser decline used a single organism, such as *Lactobacillus rhamnosus *GG [52-54]. Failure of *Lactobacillus *GG to prevent NEC in the RCT reported by Dani *et al*. and in the report of 12 years' experience by Luoto *et al*. suggests that it may be prudent to avoid the use of this single strain alone, pending further evidence [27,28,53]. The potential of *Bifidobacterium animalis *(subspecies *lactis*) also needs to be explored [58,59,76].

Using more than two or three strains (each with an optimal mass) may result in higher risk of translocation because of the substantial increase in the total dose, especially in ELBW neonates; however, without an optimal mass of each component, a combination may not be effective in assuring survival and colonisation by each strain of the supplement. It is better to avoid untested combinations, because strain combinations can be antagonistic, compatible or synergistic [77].

It would be reasonable to use probiotic products that have previously been shown to be effective in RCTs, provided the evidence indicates that there has been no change or compromise in the manufacturing technique [55,56].

### Dose

An optimal mass or dose is essential for any probiotic strain to survive and colonise the gut. The concept of viability refers to the ability of the probiotic strain to survive and proliferate in 'adequate' numbers to benefit the host. It is hence expected that there will be an optimal dose below which benefits may not occur, as survival and proliferation to adequate numbers, after overcoming the barriers such as gastric acid, bile and competing flora, is not ensured [78,79]. Evidence indicates that to be functional, probiotics have to be viable and in sufficient dosage levels, typically 10^6 ^to 10^7 ^colony-forming units (cfu)/g of product [46,80,81].

Conventional dose-response studies could be conducted in preterm neonates; however the selected doses will be arbitrary, and only guessed from what is known about the gut ecosystem in preterm neonates. There is no data on the toxic or lethal dose of probiotics for preterm neonates, and extrapolating from studies in other populations and animal experiments is likely to be incorrect [82]. An expert consensus report stated that '...there is no standardised number of probiotic bacteria that would ensure an effect [75]. The effective quantity, for a given effect and a given strain, is the quantity which has demonstrated an effect in the relevant human intervention trial'. In addition, live probiotics have the potential to replicate in the gut and lead to bacteremia. Judicious consideration is hence important in applying the principle of dose-response studies to this high-risk population with associated poor nutrition, impaired immune status and frequent exposure to infectious agents [83]. Conducting crossover and forced-titration (stepwise dose-escalation) dose-response studies will also be difficult, as the incidence of NEC is known to fluctuate over time. As for parallel design, the definition of a target dose is subjective.

Based on the median dose used in the RCTs in preterm neonates (Table 2), we suggest that a daily dose of 3 × 10^9 ^cfu/day may be appropriate for neonates of less than 32 weeks gestation. Currently, there are no data available regarding a dose beyond which the risk of probiotic complications will be high in ELBW neonates. Until such data are available, we suggest that the starting dose should be 1.5 × 10^9 ^cfu/day for ELBW neonates until they reach enteral feeds of 50 to 60 ml/kg/day. Halving the volume of the probiotic supplement should also benefit these neonates because they are often intolerant to large enteral volumes [84]. The reduced dose is still expected to be beneficial, based on the lower clinically effective doses used in the trials in our updated meta-analysis [55].

Investigators of one recent trial suggested that the daily probiotic dose in malnourished children should preferably be given as a single rather than divided dose, in view of the rapid decline of the strain mass *in vivo *[85]. The osmotic load, pH and volume of a single dose are crucial in ELBW neonates because of their inability to tolerate even very small volumes of milk feeds in the early days of life [86]. The nature of diluent (dextrose, sterile water, saline, milk) and volume after dilution are also important practical issues. The currently recommended range of osmolarity of neonatal milk formulae is 246 to 320 mOsm/kg [87]. The osmotic load of drugs and milk additives is a concern in high-risk neonates because of the risk of NEC [88-90]. Adequate dilution is thus necessary to avoid undue hyperosmolarity.

### When to start?

Because of the importance of early establishment of commensal flora in preterm neonates [40,41,91], the probiotic supplementation should be started as early as possible before pathogens colonise or antibiotics destroy the prevailing commensals. The earliest reported age at start of supplementation was 4 hours of life, in the study by Satoh *et al*. [15]. Otherwise most of the investigators assessed (7/11) started the supplementation when the neonates were ready for enteral feeds (Table 2). Clinical stability (for example, no sepsis, patent ductus arteriosus, inotropes or ileus) is desirable to ensure that the gut function has recovered after the initial illness, with minimal risk of intolerance or translocation. The optimal protocol for probiotic administration in ELBW neonates with intrauterine growth restriction needs to be confirmed [92].

### When to stop?

It is well known from animal and human (both adults and children) studies that shedding of probiotic organisms in the stool commonly stops about 2 to 3 weeks after the probiotic supplement is stopped [48,69,78,93]. Hence continued administration is necessary to promote sustained colonisation in preterm neonates until evidence is available for this high-risk population. Based on the published trials (Table 2) and the inverse relation of gestational age with NEC and all-cause mortality, it seems appropriate that supplementation could be stopped after reaching the corrected gestational age of 36 to 37 weeks, when the risk of these adverse outcomes is minimal.

### Supplementation in the presence of potentially compromised gut integrity

The risk of probiotic translocation and sepsis is higher in critically ill and/or extremely preterm neonates with potentially compromised gut integrity [94-98], and may be higher in the presence of high doses of a single strain. The current evidence is inadequate to make clear recommendations in this area [14]. Investigators reported increased mortality in recipients of probiotic (compared with placebo) in an RCT involving adults with acute pancreatitis [99]. These findings may relate to non-occlusive mesenteric ischemia in critical illness, which is exacerbated by the added bacterial load itself or a pro-inflammatory response by gut epithelial cells [100]. Extrapolating these findings to critically ill and/or extremely preterm neonates may not be appropriate, but stopping the supplementation during an acute illness (for example, proven or suspected sepsis, NEC, perinatal asphyxia) may be in the best interest of the child, pending further evidence [101]. Studies are needed to identify the optimal use of probiotics in such neonates.

### Clinical monitoring during supplementation

Intolerance (higher osmotic load causing abdominal distension, diarrhea or vomiting), probiotic sepsis and AEs (flatulence, loose stools) of additives such as prebiotic oligosaccharides need to be monitored [102]. However, the significant overlap of features of ileus of prematurity, sepsis and NEC is expected to make this issue very difficult. Frequent clinical examinations and a cautious approach are desirable until enough experience is obtained with a probiotic product and protocol in this high-risk cohort.

### Ongoing laboratory surveillance for safety

On-site expert microbiological support is vital for independent taxonomy confirmation, exclusion of contaminants and confirmation of colony counts in the reconstituted product. Microbiology laboratories should ensure that their culture media are capable of recovering the constituent bacterial species, especially at low inoculums from sterile sites. Additionally, they should be familiar with the Gram stain and phenotypic appearances of the probiotics in different media, and be aware of the possible need for extended incubation times in anaerobic conditions. In the few published reports of bacteraemia with probiotics, there is scant detail about the blood culture manufacturer or system or the media used [103-106]. Clinical isolates should be compared with probiotic strains using molecular methods such as 16S rRNA sequencing and pulsed-field gel electrophoresis [107]. The possibility of cross-contamination, resulting in nosocomial acquisition of probiotic strains by other children in the neonatal unit, should not be forgotten. Kitajima *et al*. reported colonisation rates of 73% and 91% in their probiotic group versus 12% and 44% in the control group neonates at 2 and 6 weeks respectively [52]. Costeloe *et al*. reported cross-contamination rates of 35% in their pilot clinical trial. This possibility needs to be discussed with the parents of the children in neonatal units providing probiotic supplementation. It is important for researchers to note that cross-contamination in the control arm in an RCT is expected to underestimate the true effects of probiotics [108]. Antibiotic susceptibility testing of probiotics by standardised methods should be undertaken to provide local guidance for empiric antibiotic prescribing [103-107,109,110]. The frequency of *in vivo *transfer of antibiotic-resistance mechanisms is currently unknown. The role of routine fecal surveillance cultures to detect such transfer is also unknown, and is likely to be beyond the scope of routine laboratories. Other important issues are the stability of the probiotic on transport and shelf storage, ability of the laboratory to rapidly detect probiotic sepsis, and surveillance for the development of antibiotic resistance. Regular random stool cultures are beneficial but need extra resources. Compared with lactobacilli, culturing bifidobacteria is difficult as it requires special media and expertise [111]. The rarity of bifidobacterial sepsis in the literature could relate to failure to isolate these strains in blood culture by particular techniques. Newer non-culture methods are a better option. Extensive ongoing microbiological monitoring may not be necessary if the safety and quality (from manufacturing, transport and storage on-site to use in the neonatal unit) of the probiotic product is ensured [82,112,113].

### Practical issues

Variations in the manufacturing process can significantly alter the properties of probiotic strains [51,112,114]. Variations between batches in the quality of dietary supplements are also known to occur [115]. Assurance of good manufacturing practices is thus important [116]. The choice of the packaging material plays an important role in maintaining the viability of the probiotic strains at sufficiently high levels to ensure their therapeutic activity throughout shelf life. Probiotics, by current definition, are live microorganisms that survive in the anaerobic environment of the gut, and are sensitive to oxygen, moisture and heat. Their production and packaging should therefore involve limiting their exposure to oxygen by using barrier packages and eliminating oxygen by flushing with nitrogen. The support compounds should have minimal moisture. Refrigeration is important to protect the product from significant temperature fluctuations. The product format (dry powder, sachets, ready-to-use liquid, capsules, tablets) is an important issue, as we have recently reported poor viability of strains in probiotic tablets [117].

Based on the current understanding that viability (ability to survive, proliferate and benefit the host) is an important property of probiotic strains, the proportion of viable strains in a probiotic product will be an essential determinant of its clinical efficacy. This necessitates a high degree of stringency in the manufacturing process, as required by regulatory agencies. However, evidence indicates that dead or inactivated probiotic strains, or even their cellular components and culture broths, can still have beneficial effects [118-120]. If further clinical research provides evidence to this effect, the proportion of viable strains in a probiotic product may not be a crucial issue. However, it is important to note that even if viability of the strains does not turn out to be a crucial issue in the future, the level of stringency required in the manufacturing process cannot be compromised, because there are other important issues involved, such as taxonomy confirmation and contamination. Wastage after administration of a small dose, and stability and contamination of the leftover dose are also practical issues, and availability of a product in different strengths may solve this problem. Assurance of regular supply and ready availability of a standby product is important in view of the ongoing need for routine use and research, and prevention of inflation in pricing due to the monopoly of one product.

### Role of prebiotics in probiotic products

The coexistence of probiotics and prebiotics, as found in human breast milk, is known to be synergistic [121,122]. Prebiotics have been shown to enhance the survival of endogenous probiotic organisms [123,124]. Further research, such as RCTs of probiotics versus synbiotics, is necessary to evaluate whether addition of prebiotics improves the survival and/or efficacy of probiotic strains in preterm neonates [125].

### Regulatory issues

There has been a poor track record of QC of some commercially available products [126-128], thus improvisation and standardisation of the regulatory guidelines is urgently needed. The first option involves the central regulatory agencies (for example, in Australia, this is the Therapeutic Goods Administration (TGA)) taking the responsibility of approving the QC and quality assurance (QA) practices in the manufacturing plant, and facilitating the development of a central QC laboratory for providing national backup services for independent ongoing confirmation of quality. However, this option runs the risks of administrative delays, overburdening of the central laboratory, and complete dependency of all neonatal units on its services. The second option involves development of a central QC laboratory for each state to supervise or assist the routine use of probiotics in the state neonatal units. The third option is for each institution to develop its own on-site expertise within the federal regulatory guidelines. In countries such as the USA, where probiotics ('intended to use to diagnose, cure, mitigate, treat or prevent disease and affecting structure or function of the body') are registered as drugs rather than food supplements, the regulatory restrictions on the access to probiotics will be considerable [129,130]. Substantial delay in access to probiotics is inevitable in such countries if phase I, II and III studies are to be conducted before probiotics can be made easily available [131,132]. Defining probiotics as 'foods for specialised health use' as in Japan may overcome these difficulties [133,134]. It is important to note that, although the regulatory restrictions will be more stringent if probiotics are regulated as drugs, the regulations will then at least be clear and consistently applied, and once licensed, probiotics will potentially be more accessible to consumers and physicians. Thus, in the longer term it may actually be in the patients' interest for probiotics to be regulated as drugs under some circumstances.

We believe that with cooperation between government, industry, scientists, and the International Probiotics Association, any one of these strategies could be easily adopted to increase the availability of high-quality probiotics if there is a political will to do so.

### Other potentially useful strategies

Owing to the development of aberrant gut flora and delayed colonisation by normal commensal strains in preterm neonates, early preferential feeding with breast milk and minimising exposure to antibiotics are crucial to optimise the benefits of probiotic supplementation [52]. Neonates given antibiotics at birth have been reported to retain abnormal microbial flora 4 weeks later, indicating the damaging effect of these agents [135]. Strategies for preventing sepsis are also crucial in optimising the benefits of probiotic supplementation, as sepsis needs treatment with antibiotics (anti-probiotics) [136]. The benefits of a standardised feeding protocol must not be forgotten if prevention of NEC and facilitation of enteral nutrition is the goal [137-142]. Such a protocol will help in evaluation of the efficacy of probiotics in presence of different feeding policies. For neonatal units with donor milk banks, the effect of pasteurisation on breast-milk probiotics needs to be studied, given the thermal sensitivity of probiotic strains [143]. Breast-milk oligosaccharides are not affected by pasteurisation [144].

#### Data monitoring

Probiotic supplementation is a new development in neonatal intensive care. Hence, high-quality data monitoring is essential to evaluate population outcomes in this high-risk cohort. Monitoring data during routine use is similar to post-marketing surveillance, which has a higher rate of detection of AEs (including rare ones) [145], and is helpful in comparing the benefits and risks in different populations with different management practices. Such data are essential to evaluate the effects of the intervention at a local level, and for planning future research. It is often a requirement of regulatory agencies such as the TGA when an unlicensed drug is used. The need for post-marketing surveillance has been emphasised by expert committees [146]. Collaboration between regional neonatal networks is crucial for linkage of databases.

### Information for parents

Based on the current evidence, parents are unlikely to refuse probiotics, an intervention that substantially reduces the incidence of death and life-threatening diseases such as NEC [11,14]. Because of the lack of significant experience with probiotics, especially in extremely preterm neonates, and the currently unanswered questions surrounding this intervention, it is important to ensure that parents are well informed about the benefits and potential AEs, both short- and long-term. Honesty, clarity and transparency in sharing information with the parents, and respect for their autonomy are crucial. Informed consent may be required until sufficient experience has been obtained to provide probiotics as a routine therapy without hesitation. Continued vigilance, equivalent to post-marketing surveillance, and uniform reporting are necessary to gain more data and confidence with probiotic supplementation.

### Role of placebo-controlled trials

The sum of the current evidence supports our view that the role of placebo-controlled trials is necessary only for the evaluation of new strains. From the purist's point of view, a large, definitive, placebo-controlled trial may be justified for ELBW neonates in a setting of low baseline risk, but given the current evidence and the difficulties in obtaining fully informed consent from parents, successful completion of such a trial in a realistic time frame will be difficult. We have pointed out that the issue of reproducibility in different settings has been addressed adequately. Placebo-controlled trials are not justified purely for evaluating the frequency and consequences of cross-contamination. Allowing access to a known, clinically effective, probiotic product also cannot be the justification for such a trial, especially when special regulatory schemes allow access to a life-saving intervention. For addressing other important issues such as defining the optimum intervention (which probiotic(s), what dose and timing), and assessing microbial adaptations and ecological consequences, interactions with other preventive interventions and the effect of probiotics on early development, other types of study designs such as head to head trials (comparing different products or protocols), cluster randomised and factorial trials, cohort studies and long-term follow-up studies are more suitable than placebo-controlled trials. The frequency of cross-contamination in the placebo arm of a RCT is important in this context. As for understanding the mechanisms of the benefits of probiotics in the prevention of NEC, it is important to note that the pathogenesis of NEC remains poorly understood despite extensive research for over three decades and that there are multiple pathways by which probiotic(s) can provide benefit [63]. There is a wide range of possible mechanisms that need further investigation, and several clinical observations that cannot be satisfactorily explained at the cellular level [75]. A large number of the mechanisms cannot be measured easily in humans for ethical or feasibility reasons (for example, access to tissue specimens).

### Advancing knowledge by further research while not denying probiotics to preterm neonates

High-quality definitive RCTs comparing issues such as low versus high doses, single versus multiple strains, live versus killed probiotic organisms [118,147], whole probiotics versus probiotic components [148,149], probiotics versus prebiotics, probiotics versus synbiotics, commencing supplementation 'very early' (starting on day 1 of life if the severity of initial illness is not restrictive) versus starting 'as early as possible' (ready for enteral feeds), and 'enteral plus topical' (oral spray) versus 'only enteral' supplementation, will advance the knowledge in this area. A clear understanding of the benefits and risks of probiotics will also be facilitated by the advantages of prospective and robust data collection during such research. Long-term issues such as NDI, development of allergy, sensitisation and altered immune responses also need to be monitored. The significance of exposure of preterm neonates to lactose, dextrin and cornstarch, which are used as carriers or substrates in probiotic products, needs to be evaluated [150].

### Accessing probiotic products for research versus routine use

Accessing a probiotic product (Table 2) may be relatively easy in research rather than routine use, at least until the regulatory issues are clarified. In Australia, importing a probiotic is possible with clinical trial notification approval from the TGA and a licence to import a biological product from the Australian Quarantine and Inspection Services. It is also possible in Australia, with the local approval of the Drug and Therapeutics Committee and endorsement by the TGA of named clinicians as authorised prescribers, to obtain a probiotic under a special access scheme. A similar scheme is possible in the UK. For a new product or strain, a very thorough independent QA/QC process is needed before using it in this high-risk population. Small placebo-controlled trials (rather than observational studies) will be important to rigorously assess and confirm the ability of the new strains to colonise the preterm gut if the product is to be adopted for routine use. Even minor variations in the manufacturing process can compromise the safety and efficacy of the product [51,67,112,151].

## Conclusion

We have provided evidence-based guidelines (Table 3; Table 4) for the use of probiotics in preterm neonates, as we believe that the current evidence justifies routine use of this intervention [18]. These guidelines will also be helpful for optimal use of probiotics in research settings. We believe that probiotics should be offered routinely to all high-risk preterm neonates, taking into account the unaddressed issues. The best way forward could be to offer these products routinely but still within a research framework to cover the current gaps in knowledge [28]. It is important to note that most of the unaddressed issues can be easily resolved by studies not requiring a placebo.

**Table 3:** Specific recommendations for major clinical decisions

Specific recommendations		**LOE**^**a**^**[reference]**
Selection of strains	Combination containing *Lactobacillus *and at least one *Bifidobacterium *species is preferable. *Lactobacillus *GG alone may not be effective	I [14]; II [55,56,60]; III-[3,15]

Dose	3 × 10^9 ^organisms per day, preferably in a single dose	I [14]; II [55,56,60]

When to start?	When the neonate is ready for enteral feeds, preferably within first 7 days of life	I [14]; II [55,56,60]; III [3,15]

How long to continue?	At least until 35 weeks corrected age, or discharge	II [55,56,60]

Supplementation during acute illness	Stopping the supplementation during an acute illness such as sepsis, NEC^**b**^or perinatal asphyxia may be safe	IV [94-98]

^a^Level of evidence.

^b^Necrotising enterocolitis.

**Table 4:** Guidelines for other clinical and non-clinical issues^a^

Guidelines	References
1. Starting dose for ELBW^**b**^neonates: 1.5 × 10^9 ^cfu/day^**c **^until reaching 50-60 ml/kg/day feeds	[84] and authors' opinion

2. Osmotic load: solution should be diluted to keep the osmolality below 600 mOsm/L	[86,87]

3. Diluent: sterile water or breast milk	Authors' opinion

4. Volume for administration: 1 to 1.5 ml per dose	[86] and authors' opinion

5. Clinical monitoring: patients should be monitored for intolerance (abdominal distension, diarrhea, vomiting), probiotic sepsis, and adverse effects (flatulence, loose stools) of additives such as prebiotic oligosaccharides.	[95-101] and manufacturer recommendation

6. Ongoing laboratory surveillance: Expertise in taxonomy confirmation (16S rRNA sequencing and PFGE**^d^**), ruling out contaminants, recovering probiotic strains at low inoculums from sterile sites, familiarity with the Gram stain and phenotypic appearance of probiotics, and monitoring for antibiotic susceptibility/resistance and cross-contamination are crucial.	[107]

7. Cold chain: maintenance of cold chain should be checked. Refrigerate at 4 to 10°C	Manufacturer recommendation

8. Product stability: stability should be checked by regular microbiological tests	[51,67,112,151]

9. Leftover solution should be discarded after giving small doses as it may get contaminated	Manufacturer recommendation

10. Regulatory issues: importing may be easier for research than for clinical use. National regulations on drugs and food supplements and customs quarantine guidelines should be checked	[131,132]

11. Data monitoring: high-quality data monitoring and collaboration between regional neonatal networks is crucial for monitoring outcomes at a population level	[145,146]

12. Information for parents: parents should be kept well informed about benefits and adverse effects, including the possibility of cross-contamination	[18,24]

13. Other potentially useful strategies: early preferential use of breast milk, strategies for prevention of sepsis, standardised feeding protocols, avoidance of undue prolonged exposure to antibiotic	[137-142]

**^a^**Level of evidence was applicable to specific recommendations for clinical issues (Table 3) and not to other guideline components discussed in Table 4 above.

**^b^**Extremely low birth weight.

**^c^**CFU: Colony forming units.

**^d^**Pulsed-field gel electrophoresis.

The benefits of probiotics may not be dramatic in neonatal units with a low incidence of all-cause mortality and definite NEC in preterm neonates for various reasons. Investigators have suggested that nutritional outcomes may be appropriate for probiotic research in such neonatal units, because of the beneficial effects of probiotics on the gastrointestinal tract [9,14,62,136-138].

Current evidence is inadequate in some areas of probiotic supplementation. We have erred on the side of safety in suggesting guidelines in these areas, taking into consideration the basic principle: first, do no harm. We wish to emphasise that 'routine' does not equate to 'blind' use of probiotics, a potentially powerful but double-edged weapon in this high-risk population [19]. As the debate around routine use of probiotics in preterm neonates continues, countries such as Denmark have already issued guidelines for use of probiotics in preterm neonates [152]. If prevention of death and disease and facilitation of nutrition is the goal, relying on a package of potentially better practices rather than on probiotics alone is essential [153,154].

## Competing interests

The authors declare that they have no competing interests.

## Authors' contributions

Dr D participated in the literature search, selected relevant papers and contributed to writing the manuscript. Dr R also conducted an independent literature search, selected relevant papers and contributed to writing the manuscript. Dr D and Dr R applied the levels of evidence independently and finalised them together, and resolved inconsistencies with discussion. Dr P was responsible for the concept, design and writing of the final version of the manuscript, which was seen and approved by all authors. Dr AK is an expert microbiologist, and contributed towards the relevant sections of the manuscript.

## Pre-publication history

The pre-publication history for this paper can be accessed here:

http://www.biomedcentral.com/1741-7015/9/92/prepub

## Supplementary Material

Additional file 1 **Appendix I - PubMed search results**. This appendix includes the results of PubMed (1966 to October 2010) search. Click here for file

Additional file 2 **Appendix I - EMBASE search results**. This appendix includes the results of the EMBASE (1980 to October 2010) search. Click here for file
