# Supplementary material for: Evidence-based guidelines for use of probiotics in preterm neonates
Source: BMC Med. 2011 Aug 2;9:92. doi: 10.1186/1741-7015-9-92 (PMC3163616; doi:10.1186/1741-7015-9-92)
Supplement: Additional file 1 — Appendix I - PubMed search results. This appendix includes the results of PubMed (1966 to October 2010) search. [file 1741-7015-9-92-S1.DOC]

**Appendix I: PubMed search results**

| **MeSH word** | **Yield from** **initial search** | **Relevant articles** |
| --- | --- | --- |
| 1)"Probiotics"[Mesh] AND "Culture Techniques"[Mesh] | 58 | 32 |
| 2)"Probiotics"[Mesh] AND "Classification"[Mesh] | 56 | 34 |
| 3)"Probiotics"[Mesh] AND "Bacterial Translocation"[Mesh] | 108 | 65 |
| 4)"Probiotics"[Mesh] AND "Sepsis"[Mesh] | 86 | 52 |
| 5)"Probiotics"[Mesh] AND "Informed Consent"[Mesh] OR "Consent Forms"[Mesh]) | 1 | 1 |
| 6)"Probiotics"[Mesh] AND "Legislation, Drug"[Mesh] | 14 | 10 |
| 7)"Probiotics"[Mesh] AND ("Ethics"[Mesh] OR "Ethics Committees"[Mesh] OR "Ethics Committees, Clinical"[Mesh] OR "Codes of Ethics"[Mesh] OR "Ethics Committees, Research"[Mesh] OR "Ethics, Clinical"[Mesh] OR "Ethics, Professional"[Mesh] OR "Ethics, Medical"[Mesh] OR "Bioethics"[Mesh]) | 10 | 3 |
| 8)"Probiotics"[Mesh] AND Refrigeration"[Mesh] | 9 | 9 |
| 9)"Probiotics"[Mesh] AND "Quality Control"[Mesh] | 27 | 2 |
| 10) "Probiotics"[Mesh] AND "Quality Assurance, Health Care"[Mesh] | 66 | 0 |

**Appendix 1: Search strategy details and references:**

**1) "Probiotics"[Mesh] AND "Culture Techniques"[Mesh]**

1. Kiviharju K, Leisola M, Eerikäinen T: Optimization of a Bifidobacterium longum production process*. J Biotechnol* 2005, **117**:299-308.

2. Strompfová V, Lauková A, Marcinákova M, Vasilková Z: Testing of probiotic and bacteriocin-producing lactic acid bacteria towards Eimeria sp. *Pol J Vet Sci* 2010,**13**:389-391.

3. Jin H, Higashikawa F, Noda M, Zhao X, Matoba Y, Kumagai T, Sugiyama M: Establishment of an in vitro Peyer's patch cell culture system correlative to in vivo study using intestine and screening of lactic acid bacteria enhancing intestinal immunity. *Biol Pharm Bul* 2010, **33**:289-293.

4. Gaudana SB, Dhanani AS, Bagchi T: Probiotic attributes of Lactobacillus strains isolated from food and of human origin. *Br J Nutr* 2010,**103**:1620-1628.

5. van Hoffen E, Korthagen NM, de Kivit S, Schouten B, Bardoel B, Duivelshof A, Knol J, Garssen J, Willemsen LE: Exposure of intestinal epithelial cells to UV-killed Lactobacillus GG but not Bifidobacterium breve enhances the effector immune response in vitro*. Int Arch Allergy Immunol* 2010,**52**:159-168.

6. Moussavi M, Adams MC: An in vitro study on bacterial growth interactions and intestinal epithelial cell adhesion characteristics of probiotic combinations. *Curr Microbiol* 2010,**60**:327-335.

7. de Roock S, van Elk M, van Dijk ME, Timmerman HM, Rijkers GT, Prakken BJ,

Hoekstra MO, de Kleer IM: Lactic acid bacteria differ in their ability to induce functional regulatory T cells in humans. *Clin Exp Allergy* 2010,**40**:103-110.

8. Liu SQ, Tsao M: Enhancement of survival of probiotic and non-probiotic lactic acid bacteria by yeasts in fermented milk under non-refrigerated conditions. *Int J Food Microbiol* 2009,**135**:34-38.

9. Hagi T, Hoshino T: Screening and characterization of potential probiotic lactic acid bacteria from cultured common carp intestine. *Biosci Biotechnol Biochem* 2009,**73**:1479-1483.

10. Ruiz L, Sánchez B, de Los Reyes-Gavilán CG, Gueimonde M, Margolles A: Coculture of Bifidobacterium longum and Bifidobacterium breve alters their protein expression profiles and enzymatic activities. *Int J Food Microbiol* 2009,**133**:148-153.

11. Tabasco R, García-Cayuela T, Peláez C, Requena T: Lactobacillus acidophilus La-5 increases lactacin B production when it senses live target bacteria. *Int J Food Microbiol* 2009, **132**:109-116.

12. Oliveira RP, Florence AC, Silva RC, Perego P, Converti A, Gioielli LA, Oliveira MN: Effect of different prebiotics on the fermentation kinetics, probiotic survival and fatty acids profiles in nonfat symbiotic fermented milk. *Int J Food Microbiol* 2009,**128**:467-472.

13. Betenbaugh M, Bentley W: Metabolic engineering in the 21st century: meeting global challenges of sustainability and health. *Curr Opin Biotechnol* 2008,**19**:411-413.

14. Lin J, Yu W, Liu X, Xie H, Wang W, Ma X: In Vitro and in Vivo characterization of alginate-chitosan-alginate artificial microcapsules for therapeutic oral delivery of live bacterial cells. *J Biosci Bioeng* 2008,**105**:660-665.

15. Yang J, Huang K, Qin S, Wu X, Zhao Z, Chen F:Antibacterial action of selenium-enriched probiotics against pathogenic Escherichia coli. *Dig Dis Sci* 2009,**54**:246-254.

16. Altieri C, Bevilacqua A, D'Amato D, Nobile MA, Sinigaglia M: Modelling the survival of starter lactic acid bacteria and Bifidobacterium bifidum in single and simultaneous cultures. *Food Microbiol* 2008,**25**:729-734.

17. Lavermicocca P, Valerio F, Lonigro SL, Di Leo A, Visconti A: Antagonistic activity of potential probiotic Lactobacilli against the ureolytic pathogen Yersinia enterocolitica. *Curr Microbiol* 2008, **56**:175-181.

18. Cepeljnik T, Lah B, Narat M, Marinsek-Logar R: Adaptation of adhesion test using Caco-2 cells for anaerobic bacterium Pseudobutyrivibrio xylanivorans, a probiotic candidate. *Folia Microbiol (Praha)* 2007,**52**:367-373.

19. Yang F, Wang J, Li X, Ying T, Qiao S, Li D, Wu G: 2-DE and MS analysis of interactions between Lactobacillus fermentum I5007 and intestinal epithelial cells. *Electrophoresis* 2007,**28**:4330-4339.

20. Dehlink E, Domig KJ, Loibichler C, Kampl E, Eiwegger T, Georgopoulos A, Kneifel W, Urbanek R, Szépfalusi Z: Heat- and formalin-inactivated probiotic bacteria induce comparable cytokine patterns in intestinal epithelial cell-leucocyte cocultures. *J Food Prot* 2007,**70**:2417-2421.

21. Timmerman HM, Niers LE, Ridwan BU, Koning CJ, Mulder L, Akkermans LM, Rombouts FM, Rijkers GT: Design of a multispecies probiotic mixture to prevent infectious complications in critically ill patients. *Clin Nutr* 2007,**26**:450-459.

22. Tsaruk'ianova IG, Osadchaia AI: Joint cultivation of Bacillus subtilis and Escherichia coli strains promising for obtaining complex probiotic. *Mikrobiol Z* 2007,**69**:43-49.

23. Lacroix C, Yildirim S: Fermentation technologies for the production of probiotics with high viability and functionality. *Curr Opin Biotechnol* 2007,**18**:176-183.

24. Buriti FC, Cardarelli HR, Saad SM: Biopreservation by Lactobacillus paracasei in coculture with Streptococcus thermophilus in potentially probiotic and synbiotic fresh cream cheeses. *J Food Prot* 2007,**70**:228-235.

25. Aslim B, Onal D, Beyatli Y: Factors influencing autoaggregation and aggregation of Lactobacillus delbrueckii subsp. bulgaricus isolated from handmade yogurt. *J Food Prot* 2007,**70**:223-227.

26. Yan F, Cao H, Cover TL, Whitehead R, Washington MK, Polk DB: Soluble proteins produced by probiotic bacteria regulate intestinal epithelial cell survival and growth. *Gastroenterology* 2007,**132**:562-575.

27. Schellenberg J, Smoragiewicz W, Karska-Wysocki B: A rapid method combining immunofluorescence and flow cytometry for improved understanding of competitive interactions between lactic acid bacteria (LAB) and methicillin-resistant S. aureus (MRSA) in mixed culture. *J Microbiol Methods* 2006,**65**:1-9.

28. Glushanova NA, Shenderov BA: Relationships between the probiotic and host indigenous lactobacilli under the conditions of mixed cultivation in vitro]. *Zh Mikrobiol Epidemiol Immunobiol* 2005,**2**:56-61.

29. van der Aa Kühle A, Skovgaard K, Jespersen L: In vitro screening of probiotic properties of Saccharomyces cerevisiae var. boulardii and food-borne Saccharomyces cerevisiae strains. *Int J Food Microbiol* 2005,**101**:29-39.

30. Shiba T, Aiba Y, Ishikawa H, Ushiyama A, Takagi A, Mine T, Koga Y: The suppressive effect of bifidobacteria on Bacteroides vulgatus, a putative pathogenic microbe in inflammatory bowel disease. *Microbiol Immunol* 2003,**47**:371-378.

31. Giese T, Zimmermann K, Meuer SC: Functional characterization of pro-biotic pharmaceuticals by quantitative analysis of gene expression. *Arzneimittelforschung* 2003,**53**:385-391.

32. Hosoi T, Ametani A, Kiuchi K, Kaminogawa S: Improved growth and viability of lactobacilli in the presence of Bacillus subtilis (natto), catalase, or subtilisin. *Can J Microbiol* 2000,**46**:892-897.

**2) "Probiotics"[Mesh] AND "Classification"[Mesh]**

1. Felis GE, Dellaglio F: Taxonomy of Lactobacilli and Bifidobacteria: *Curr Issues Intest Microbiol* 2007,**8**:44-61.

2. Coudeyras S, Marchandin H, Fajon C, Forestier C: Taxonomic and strain-specific identification of the probiotic strain Lactobacillus rhamnosus 35 within the Lactobacillus casei group. *Appl Environ Microbiol* 2008,**74**:2679-2689.

3. Lee DY, Seo YS, Rayamajhi N, Kang ML, Lee SI, Yoo HS: Isolation, characterization, and evaluation of wild isolates of Lactobacillus reuteri from pig feces. *J Microbiol* 2009,**47**:663-672.

4. Cox MJ, Huang YJ, Fujimura KE, Liu JT, McKean M, Boushey HA, Segal MR, Brodie EL, Cabana MD, Lynch SV: Lactobacillus casei abundance is associated with profound shifts in the infant gut microbiome. *PLoS One* 2010, **5**:e8745.

5. Mahenthiralingam E, Marchbank A, Drevinek P, Garaiova I, Plummer S: Use of colony-based bacterial strain typing for tracking the fate of Lactobacillus strains during human consumption. *BMC Microbiol* 2009,**9**:251.

6. Sisto A, De Bellis P, Visconti A, Morelli L, Lavermicocca P: Development of a PCR assay for the strain-specific identification of probiotic strain Lactobacillus paracasei IMPC2.1*. Int J Food Microbiol* 2009,**136**:59-65.

7. Gao P, Sun Z, Ma S, Wang Q, Gao J, Deng C, Zhang H: Screening and identification of probiotic bifidobacterium from Mongolian Children. *Wei Sheng Wu Xue Bao* 2009,**49**:210-216.

8. Yu Z, Dong B, Lu W: Dynamics of bacterial community in solid-state fermented feed revealed by 16S rRNA*. Lett Appl Microbiol* 2009,**49**:166-172.

9. Patel AK, Ahire JJ, Pawar SP, Chaudhari BL, Shouche YS, Chincholkar SB: Evaluation of probiotic characteristics of siderophoregenic Bacillus spp. isolated from dairy waste. *Appl Biochem Biotechnol* 2010,**160**:140-155.

10. Ryan KA, Jayaraman T, Daly P, Canchaya C, Curran S, Fang F, Quigley EM, O'Toole PW: Isolation of lactobacilli with probiotic properties from the human stomach. *Lett Appl Microbiol* 2008,**47**:269-274.

11. I Higuchi W, Muramatsu M, Dohmae S, Takano T, Isobe H, Yabe S, Da S, Baranovich T, Yamamoto T: Identification of probiotic lactobacilli used for animal feeds on the basis of 16S ribosomal RNA gene sequence. *Microbiol Immunol* 2008,**52**:559-563.

12. Vasquez N, Suau A, Magne F, Pochart P, Pélissier MA: Differential effects of Bifidobacterium pseudolongum strain Patronus and metronidazole in the rat gut. *Appl Environ Microbiol* 2009,**75**:381-386.

13. Yun JH, Lee KB, Sung YK, Kim EB, Lee HG, Choi YJ: Isolation and characterization of potential probiotic lactobacilli from pig feces. *J Basic Microbiol* 2009,**49**:220-226.

14.Sidorenko AV, Novik GI, Akimov VN: Application of the methods of molecular systematics to classification and identification of bacteria of the genus ifidobacterium. *Mikrobiologiia* 2008,**77**:293-302.

15. MacKenzie DA, Defernez M, Dunn WB, Brown M, Fuller LJ, de Herrera SR, Günther A, James SA, Eagles J, Philo M, Goodacre R, Roberts IN: Relatedness of medically important strains of Saccharomyces cerevisiae as revealed by phylogenetics and metabolomics. *Yeast* 2008,**25**:501-512.

16. Meile L, Le Blay G, Thierry A: Safety assessment of dairy microorganisms: Propionibacterium and Bifidobacterium. *Int J Food Microbiol* 2008,**126**:316-320.

17. Bernardeau M, Vernoux JP, Henri-Dubernet S, Guéguen M: Safety assessment of dairy microorganisms: the Lactobacillus genus. *Int J Food Microbiol* 2008,**126**:278-285.

18. Ventura M, O'Connell-Motherway M, Leahy S, Moreno-Munoz JA, Fitzgerald GF, van mSinderen D: From bacterial genome to functionality; case bifidobacteria. *Int J Food Microbiol* 2007,**120**:2-12.

19. Blaut M, Clavel T: Metabolic diversity of the intestinal microbiota: implications for health and disease. *J Nutr* 2007,**137**(3 Suppl 2):751S-755S.

20. Nakanishi Y, Murashima K, Ohara H, Suzuki T, Hayashi H, Sakamoto M, Fukasawa T, Kubota H, Hosono A, Kono T, Kaminogawa S, Benno Y: Increase in terminal restriction fragments of Bacteroidetes-derived 16S rRNA genes after administration of short-chain fructooligosaccharides. *Appl Environ Microbiol* 2006,**72**:6271-6276.

21. Maruo T, Sakamoto M, Toda T, Benno Y: Monitoring the cell number of Lactococcus lactis subsp. cremoris FC in human feces by real-time PCR with strain-specific primers designed using the RAPD technique. *Int J Food Microbiol* 2006,1:**110**:69-76.

22. Bondarenko VM: Molecular-and-genetic and molecular-and-biological studies of Bifidobacterium and Lactobacillus representatives. *Vestn Ross Akad Med Nauk* 2006,**1**:18-24.

23. Simpson PJ, Stanton C, Fitzgerald GF, Ross RP: Intrinsic tolerance of Bifidobacterium species to heat and oxygen and survival following spray drying and storage. *J Appl Microbiol* 2005,**99**:493-501.

24. Posteraro B, Sanguinetti M, Romano L, Torelli R, Novarese L, Fadda G: Molecular tools for differentiating probiotic and clinical strains of Saccharomyces cerevisiae. *Int J Food Microbiol* 2005,**103**:295-304.

25. Hjelm M, Bergh O, Riaza A, Nielsen J, Melchiorsen J, Jensen S, Duncan H, Ahrens P, Birkbeck H, Gram L: Selection and identification of autochthonous potential probiotic bacteria from turbot larvae (Scophthalmus maximus) rearing units. *Syst Appl Microbiol* 2004,**27**:360-371.

26. Schillinger U, Yousif NM, Sesar L, Franz CM: Use of group-specific and RAPD-PCR analyses for rapid differentiation of Lactobacillus strains from probiotic yogurts. *Curr Microbiol* 2003,**47**:453-456.

27. Masco L, Huys G, Gevers D, Verbrugghen L, Swings J: Identification of Bifidobacterium species using rep-PCR fingerprinting. *Syst Appl Microbiol* 2003,**26**:557-563.

28. Klein G: Use of molecular methods in food microbiology with the example of probiotic use of lactobacilli. *Berl Munch Tierarztl Wochenschr* 2003,**116**:510-516.

29. Klein G: Taxonomy, ecology and antibiotic resistance of enterococci from food and the gastro-intestinal tract. *Int J Food Microbiol* 2003,**88:**123-131.

30. Franz CM, Stiles ME, Schleifer KH, Holzapfel WH: Enterococci in foods--a conundrum for food safety. *Int J Food Microbiol* 2003,**88**:105-122.

31. Suau A: Molecular tools to investigate intestinal bacterial communities. *J Pediatr Gastroenterol Nutr* 2003,**37**:222-224.

32. Delley M, Germond JE: Differentiation of Lactobacillus helveticus, Lactobacillus delbrueckii subsp bulgaricus, subsp lactis and subsp delbrueckii using physiological and genetic tools and reclassification of some strains from the ATCC collection. *Syst Appl Microbiol* 2002,**25**:228-231.

33. Roy D, Sirois S, Vincent D: Molecular discrimination of lactobacilli used as starter and probiotic cultures by amplified ribosomal DNA restriction analysis. *Curr Microbiol* 2001,**42**:282-289.

34. Hoa NT, Baccigalupi L, Huxham A, Smertenko A, Van PH, Ammendola S, Ricca E, Cutting AS: Characterization of Bacillus species used for oral bacteriotherapy and

bacterioprophylaxis of gastrointestinal disorders. *Appl Environ Microbiol* 2000,**66**:5241-5247.

**3) "Probiotics"[Mesh] AND "Bacterial Translocation"[Mesh]**

1. Liong MT: Safety of probiotics: translocation and infection. *Nutr Rev* 2008,**66**:192-202.

2. Generoso SV, Viana M, Santos R, Martins FS, Machado JA, Arantes RM, Nicoli JR, Correia MI, Cardoso VN: Saccharomyces cerevisiae strain UFMG 905 protects against bacterial translocation, preserves gut barrier integrity and stimulates the immune system in a murine intestinal obstruction model. *Arch Microbiol* 2010,**192**:477-484.

3. Cukrowska B, Motyl I, Kozáková H, Schwarzer M, Górecki RK, Klewicka E, Slizewska K, Libudzisz Z: Probiotic Lactobacillus strains: in vitro and in vivo studies. *Folia Microbiol (Praha)* 2009,**54**:533-537.

4. Wu J, Wang X, Cai W, Hong L, Tang Q: Bifidobacterium adolescentis supplementation ameliorates parenteral nutrition-induced liver injury in infant rabbits. *Dig Dis Sci* 2010,**55**:2814-2820.

5. Li YT, Wang L, Chen Y, Chen YB, Wang HY, Wu ZW, Li LJ: Effects of gut microflora on hepatic damage after acute liver injury in rats. *J Trauma* 2010,**68**:76-83.

6. Besselink MG, van Santvoort HC, Renooij W, de Smet MB, Boermeester MA, Fischer K, Timmerman HM, Ahmed Ali U, Cirkel GA, Bollen TL, van Ramshorst B, Schaapherder AF, Witteman BJ, Ploeg RJ, van Goor H, van Laarhoven CJ, Tan AC, Brink MA, van der Harst E, Wahab PJ, van Eijck CH, Dejong CH, van Erpecum KJ, Akkermans LM, Gooszen HG: Dutch Acute Pancreatitis Study Group. Intestinal barrier dysfunction in a randomized trial of a specific probiotic composition in acute pancreatitis. *Ann Surg* 2009,**250**:712-719.

7. Abe F, Muto M, Yaeshima T, Iwatsuki K, Aihara H, Ohashi Y, Fujisawa T: Safety evaluation of probiotic bifidobacteria by analysis of mucin degradation activity and translocation ability. *Anaerobe* 2010,**16**:131-136.

8.Jain S, Yadav H, Sinha PR: Probiotic dahi containing Lactobacillus casei protects against Salmonella enteritidis infection and modulates immune response in mice. *J Med Food* 2009, **12**:576-583.

9. Ruan XC, Wang SM, Shi HP, Li XX, Xia FG, Ming FP: Protective effects of micro-encapsulated Bifidobacteria on gut barrier after hemorrhagic shock and resuscitation: experiment with rats. *Zhonghua Yi Xue Za Zhi* 2009,**89**:625-629.

10. Yakabe T, Moore EL, Yokota S, Sui H, Nobuta Y, Fukao M, Palmer H, Yajima N: Safety assessment of Lactobacillus brevis KB290 as a probiotic strain. *Food Chem Toxicol* 2009,**47**:2450-2453.

11. Theodorou D, Aggeli P, Markogiannakis H, Skouroliakou M, Archontovasilis F, Kastanidou O, Burnetas A, Xiromeritou V, Katsaragakis S: Protection of intestinal permeability in the perioperative period. *J Clin Gastroenterol* 2009,**43**:500.

12. Copeland DR, McVay MR, Dassinger MS, Jackson RJ, Smith SD: Probiotic fortified diet reduces bacterial colonization and translocation in a long-term neonatal rabbit model. *J Pediatr Surg* 2009,**44**:1061-1064.

13. Karen M, Yuksel O, Akyürek N, Ofluoğlu E, Cağlar K, Sahin TT, Paşaoğlu H, Memiş L, Akyürek N, Bostanci H: Probiotic agent Saccharomyces boulardii reduces the incidence of lung injury in acute necrotizing pancreatitis induced rats. *J Surg Res* 2010,**160**:139-144.

14. Schiffrin EJ, Parlesak A, Bode C, Bode JC, van't Hof MA, Grathwohl D, Guigoz Y: Probiotic yogurt in the elderly with intestinal bacterial overgrowth: endotoxaemia and innate immune functions. *Br J Nutr* 2009,**101**:961-966.

15. Boneti C, Habib CM, Keller JE, Diaz JA, Kokoska ER, Jackson RJ, Smith SD: Probiotic acidified formula in an animal model reduces pulmonary and gastric bacterial load. *J Pediatr Surg* 2009,**44**:530-533.

16. Rychter JW, van Minnen LP, Verheem A, Timmerman HM, Rijkers GT, Schipper ME, Gooszen HG, Akkermans LM, Kroese AB: Pretreatment but not treatment with probiotics abolishes mouse intestinal barrier dysfunction in acute pancreatitis. *Surgery* 2009,**145**:157-167.

17. Guerrero Hernández I, Torre Delgadillo A, Vargas Vorackova F, Uribe M: Intestinal flora, probiotics, and cirrhosis. *Ann Hepatol* 2008,**7**:120-124.

18. Paturi G, Phillips M, Kailasapathy K: Effect of probiotic strains Lactobacillus acidophilus LAFTI L10 and Lactobacillus paracasei LAFTI L26 on systemic immune functions and bacterial translocation in mice. *J Food Prot*. 2008,**71**:796-801.

19. Southcott E, Tooley KL, Howarth GS, Davidson GP, Butler RN: Yoghurts containing probiotics reduce disruption of the small intestinal barrier in methotrexate-treated rats. *Dig Dis Sci* 2008,**53**:1837-1841.

20. Laudanno OM, Cesolari JA, Godoy A, Sutich E, Sarangone S, Catalano J, San Miguel P: Bioflora probiotic in immunomodulation and prophylaxis of intestinal bacterial translocation in rats. *Dig Dis Sci* 2008,**53**:2667-2670.

21. McVay MR, Boneti C, Habib CM, Keller JE, Kokoska ER, Jackson RJ, Smith SD: Formula fortified with live probiotic culture reduces pulmonary and gastrointestinal bacterial colonization and translocation in a newborn animal model. *J Pediatr Surg* 2008,**43**:25-29.

22. Sentongo TA, Cohran V, Korff S, Sullivan C, Iyer K, Zheng X: Intestinal permeability and effects of Lactobacillus rhamnosus therapy in children with short bowel syndrome. *J Pediatr Gastroenterol Nutr* 2008,**46**:41-47.

23. Kabeir BM, Yazid AM, Stephenie W, Hakim MN, Anas OM, Shuhaimi M: Safety evaluation of Bifidobacterium pseudocatenulatum G4 as assessed in BALB/c mice. *Lett Appl Microbiol* 2008,**46**:32-37.

24. Mogilner JG, Srugo I, Lurie M, Shaoul R, Coran AG, Shiloni E, Sukhotnik I: Effect of probiotics on intestinal regrowth and bacterial translocation after massive small bowel resection in a rat. *J Pediatr Surg* 2007,**42**:1365-1371.

25. Ruan X, Shi H, Xia G, Xiao Y, Dong J, Ming F, Wang S: Encapsulated Bifidobacteria reduced bacterial translocation in rats following hemorrhagic shock and resuscitation. *Nutrition* 2007,**23**:754-761.

26. Seal M, Naito Y, Barreto R, Lorenzetti A, Safran P, Marotta F: Experimental radiotherapy-induced enteritis: a probiotic interventional study. *J Dig Dis* 2007,**8**:143-147.

27. Lara-Villoslada F, Sierra S, Díaz-Ropero MP, Olivares M, Xaus J: Safety assessment of the human milk-isolated probiotic Lactobacillus salivarius CECT5713. *J Dairy Sci* 2007,**90**:3583-3589.

28. Lara-Villoslada F, Sierra S, Martín R, Delgado S, Rodríguez JM, Olivares M, Xaus J: Safety assessment of two probiotic strains, Lactobacillus coryniformis CECT5711 and Lactobacillus gasseri CECT5714. *J Appl Microbiol* 2007, **103**:175-184.

29. van Minnen LP, Timmerman HM, Lutgendorff F, Verheem A, Harmsen W, Konstantinov SR, Smidt H, Visser MR, Rijkers GT, Gooszen HG, Akkermans LM: Modification of intestinal flora with multispecies probiotics reduces bacterial translocation and improves clinical course in a rat model of acute pancreatitis. *Surgery* 2007,**141**:470-480.

30. Gatt M, Reddy BS, MacFie J: Review article: bacterial translocation in the critically ill--evidence and methods of prevention. *Aliment Pharmacol Ther* 2007,**25**:741-757.

31. Mangell P, Lennernäs P, Wang M, Olsson C, Ahrné S, Molin G, Thorlacius H, Jeppsson B: Adhesive capability of Lactobacillus plantarum 299v is important for preventing bacterial translocation in endotoxemic rats. *APMIS* 2006,**114**:611-618.

32. Shen TY, Qin HL, Gao ZG, Fan XB, Hang XM, Jiang YQ: Influences of enteral nutrition combined with probiotics on gut microflora and barrier function of rats with abdominal infection. *World J Gastroenterol* 2006,**12**:4352-4358.

33. Zareie M, Johnson-Henry K, Jury J, Yang PC, Ngan BY, McKay DM, Soderholm JD, Perdue MH, Sherman PM: Probiotics prevent bacterial translocation and improve intestinal barrier function in rats following chronic psychological stress. *Gut* 2006, **55**:1553-1560.

34. Demirer S, Aydintug S, Aslim B, Kepenekci I, Sengül N, Evirgen O, Gerceker D, Andrieu MN, Ulusoy C, Karahüseyinoglu S: Effects of probiotics on radiation- induced intestinal injury in rats. *Nutrition* 2006,**22**:179-186.

35. Besselink MG, Timmerman HM, van Minnen LP, Akkermans LM, Gooszen HG: Prevention of infectious complications in surgical patients: potential role of probiotics. *Dig Surg* 2005,**22**:234-244.

36. Gun F, Salman T, Gurler N, Olgac V: Effect of probiotic supplementation on bacterial translocation in thermal injury. *Surg Today* 2005,**35**:760-764.

37. Luyer MD, Buurman WA, Hadfoune M, Speelmans G, Knol J, Jacobs JA, Dejong CH, Vriesema AJ, Greve JW: Strain-specific effects of probiotics on gut barrier integrity following hemorrhagic shock*. Infect Immun* 2005,**73**:3686-3692.

38. Qin HL, Shen TY, Gao ZG, Fan XB, Hang XM, Jiang YQ, Zhang HZ: Effect of lactobacillus on the gut microflora and barrier function of the rats with abdominal infection. *World J Gastroenterol* 2005,**11**:2591-2596.

39. Nikitenko VI: Infection prophylaxis of gunshot wounds using probiotics. *J Wound Care* 2004,**13**:363-366.

40. Hammerman C, Bin-Nun A, Kaplan M: Germ warfare: probiotics in defense of the premature gut. *Clin Perinatol* 2004,**31**:489-500.

41. Jain PK, McNaught CE, Anderson AD, MacFie J, Mitchell CJ: Influence of synbiotic containing Lactobacillus acidophilus La5, Bifidobacterium lactis Bb 12, Streptococcus thermophilus, Lactobacillus bulgaricus and oligofructose on gut barrier function and sepsis in critically ill patients: a randomised controlled trial. *Clin Nutr* 2004,**23**:467-475.

42. Seehofer D, Rayes N, Schiller R, Stockmann M, Müller AR, Schirmeier A, Schaeper F, Tullius SG, Bengmark S, Neuhaus P: Probiotics partly reverse increased bacterial translocation after simultaneous liver resection and colonic anastomosis in rats*. J Surg Res* 2004,**117**:262-271.

43. Herek O, Kara IG, Kaleli I: Effects of antibiotics and Saccharomyces boulardii on bacterial translocation in burn injury. *Surg Today* 2004,**34**:256-260.

44. Cano PG, Perdigón G: Probiotics induce resistance to enteropathogens in a re-nourished mouse model. *J Dairy Res* 2003,**70**:433-440.

45. Von Bültzingslöwen I, Adlerberth I, Wold AE, Dahlén G, Jontell M: Oral and intestinal microflora in 5-fluorouracil treated rats, translocation to cervical and mesenteric lymph nodes and effects of probiotic bacteria. *Oral Microbiol Immunol* 2003,**18**:278-284.

46. García-Urkia N, Asensio AB, Zubillaga Azpiroz I, Zubillaga Huici P, Vidales C,

García-Arenzana JM, Aldazábal P, Eizaguirre I: Beneficial effects of ifidobacterium lactis in the prevention of bacterial translocation in experimental short bowel syndrome*. Cir Pediatr* 2002,**15**:162-165.

47. Guarner F, Malagelada JR: Gut flora in health and disease. *Lancet* 2003,**361**:512-519.

48. McNaught CE, Woodcock NP, MacFie J, Mitchell CJ: A prospective randomised study of the probiotic Lactobacillus plantarum 299V on indices of gut barrier function in elective surgical patients. *Gut* 2002,**51**:827-831.

49. Eizaguirre I, Urkia NG, Asensio AB, Zubillaga I, Zubillaga P, Vidales C, Garcia-Arenzana JM, Aldazabal P: Probiotic supplementation reduces the risk of bacterial translocation in experimental short bowel syndrome. *J Pediatr Surg* 2002,**37**:699-702.

50. Bauer TM, Fernández J, Navasa M, Vila J, Rodés J: Failure of Lactobacillus spp. to prevent bacterial translocation in a rat model of experimental cirrhosis. *J Hepatol* 2002,**36**:501-506.

51. Gill HS, Shu Q, Lin H, Rutherfurd KJ, Cross ML: Protection against translocating Salmonella typhimurium infection in mice by feeding the immuno-enhancing probiotic Lactobacillus rhamnosus strain HN001. *Med Microbiol Immunol* 2001,**190**:97-104.

52. Adawi D, Ahrné S, Molin G: Effects of different probiotic strains of Lactobacillus and Bifidobacterium on bacterial translocation and liver injury in an acute liver injury model. *Int J Food Microbiol* 2001,**70**:213-220.

53. Rodriguez AV, Baigorí MD, Alvarez S, Castro GR, Oliver G: Phosphatidylinositol-specific phospholipase C activity in Lactobacillus rhamnosus with capacity to translocate. *FEMS Microbiol Lett* 2001,**204**:33-38.

54. Mattar AF, Drongowski RA, Coran AG, Harmon CM: Effect of probiotics on enterocyte bacterial translocation in vitro. *Pediatr Surg Int* 2001,**17**:265-268.

55. Ishibashi N, Yamazaki S: Probiotics and safety. *Am J Clin Nut* 2001,**73**(2 Suppl):S465-S470.

56. Carina Audisio M, Oliver G, Apella MC: Protective effect of Enterococcus faecium J96, a potential probiotic strain, on chicks infected with Salmonella Pullorum. *J Food Prot* 2000,**63**:1333-1337.

57. Lee DJ, Drongowski RA, Coran AG, Harmon CM: Evaluation of probiotic treatment in a neonatal animal model. *Pediatr Surg Int* 2000,**16**:237-242.

58. Shu Q, Lin H, Rutherfurd KJ, Fenwick SG, Prasad J, Gopal PK, Gill HS: Dietary Bifidobacterium lactis (HN019) enhances resistance to oral Salmonella typhimurium infection in mice. *Microbiol Immunol* 2000,**44**:213-222.

59. Mangiante G, Canepari P, Colucci G, Marinello P, Signoretto C, Nicoli N, Bengmark S: A probiotic as an antagonist of bacterial translocation in experimental pancreatitis. *Chir Ital* 1999,**51**:221-226.

60. Duffy LC: Interactions mediating bacterial translocation in the immature intestine. *J Nutr* 2000,**130**(2S Suppl):432S-436S.

61. Dai D, Walker WA: Protective nutrients and bacterial colonization in the immature human gut. *Adv Pediatr* 1999,**46**:353-82.

62. Urao M, Fujimoto T, Lane GJ, Seo G, Miyano T: Does probiotics administration decrease serum endotoxin levels in infants? *J Pediatr Surg* 1999,**34**:273-276.

63. Naaber P, Mikelsaar RH, Salminen S, Mikelsaar M: Bacterial translocation, intestinal microflora and morphological changes of intestinal mucosa in experimental models of Clostridium difficile infection. *J Med Microbiol* 1998,**47**:591-598.

64. Seehofer D, Rayes N, Schiller R, Stockmann M, Müller AR, Schirmeier A, Schaeper F, Tullius SG, Bengmark S, Neuhaus P: Probiotics partly reverse increased bacterial translocation after simultaneous liver resection and colonic anastomosis in rats. *J Surg Res* 2004,**117**:262-271.

65. Mattar AF, Drongowski RA, Coran AG, Harmon CM: Effect of probiotics on enterocyte bacterial translocation in vitro*. Pediatr Surg Int* 2001,**17**:265-268.

**4) "Probiotics"[Mesh] AND "Sepsis"[Mesh]**

1. Ohishi A, Takahashi S, Ito Y, Ohishi Y, Tsukamoto K, Nanba Y, Ito N, Kakiuchi S, Saitoh A, Morotomi M, Nakamura T: Bifidobacterium septicemia associated with postoperative probiotic therapy in a neonate with omphalocele. *J Pediatr* 2010,**156**:679-681.

2. Guenther K, Straube E, Pfister W, Guenther A, Huebler A: Severe sepsis after probiotic treatment with Escherichia coli NISSLE 1917. *Pediatr Infect Dis J* 2010,**29**:188-189.

3. Piechno S, Seguin P, Gangneux JP: Saccharomyces boulardii fungal sepsis: beware of the yeast. *Can J Anaesth* 2007,**54**:245-246.

4. Riquelme AJ, Calvo MA, Guzmán AM, Depix MS, García P, Pérez C, Arrese M, Labarca JA: Saccharomyces cerevisiae fungemia after Saccharomyces boulardii treatment in immunocompromised patients*. J Clin Gastroenterol* 2003,**36**:41-43.

5. Salminen MK, Tynkkynen S, Rautelin H, Saxelin M, Vaara M, Ruutu P, Sarna S, Valtonen V, Järvinen A: Lactobacillus bacteremia during a rapid increase in probiotic use of Lactobacillus rhamnosus GG in Finland. *Clin Infect Dis* 2002,**35**:1155-1160.

6. Berger RE: Lactobacillus sepsis associated with probiotic therapy. *J Urol* 2005,**174**:1843.

7. Kunz AN, Fairchok MP, Noel JM: Lactobacillus sepsis associated with probiotic therapy. *Pediatrics* 2005,**116**:517.

8. Herbrecht R, Nivoix Y: Saccharomyces cerevisiae fungemia: an adverse effect of Saccharomyces boulardii probiotic administration. *Clin Infect Dis* 2005,**40**:1635-1637.

9. Muñoz P, Bouza E, Cuenca-Estrella M, Eiros JM, Pérez MJ, Sánchez-Somolinos M, Rincón C, Hortal J, Peláez T: Saccharomyces cerevisiae fungemia: an emerging infectious disease. *Clin Infect Dis* 2005,**40**:1625-1634.

10. De Groote MA, Frank DN, Dowell E, Glode MP, Pace NR: Lactobacillus rhamnosus GG bacteremia associated with probiotic use in a child with short gut syndrome. *Pediatr Infect Dis J* 2005,**24**:278-280.

11. Land MH, Rouster-Stevens K, Woods CR, Cannon ML, Cnota J, Shetty AK: Lactobacillus sepsis associated with probiotic therapy. *Pediatrics* 2005,**115**:178-181.

12. Besselink MG, van Santvoort HC, Renooij W, de Smet MB, Boermeester MA, Fischer K, Timmerman HM, Ahmed Ali U, Cirkel GA, Bollen TL, van Ramshorst B, Schaapherder AF, Witteman BJ, Ploeg RJ, van Goor H, van Laarhoven CJ, Tan AC, Brink MA, van der Harst E, Wahab PJ, van Eijck CH, Dejong CH, van Erpecum KJ, Akkermans LM, Gooszen HG: Dutch Acute Pancreatitis Study Group. Intestinal barrier dysfunction in a randomized trial of a specific probiotic composition in acute pancreatitis. *Ann Surg* 2009, **250**:712-719.

13. Manzoni P, Rinaldi M, Cattani S, Pugni L, Romeo MG, Messner H, Stolfi I, Decembrino L, Laforgia N, Vagnarelli F, Memo L, Bordignon L, Saia OS, Maule M, Gallo E, Mostert M, Magnani C, Quercia M, Bollani L, Pedicino R, Renzullo L, Betta P, Mosca F, Ferrari F, Magaldi R, Stronati M, Farina D: Italian Task Force for the Study and Prevention of Neonatal Fungal Infections, Italian Society of Neonatology. Italian Society of Neonatology. Bovine lactoferrin supplementation for prevention of late-onset sepsis in very low-birth-weight neonates: a randomized trial. *JAMA* 2009,**302**:1421-1428.

14.. Arribas B, Rodríguez-Cabezas ME, Camuesco D, Comalada M, Bailón E, Utrilla P, Nieto A, Concha A, Zarzuelo A, Gálvez J: A probiotic strain of Escherichia coli, Nissle 1917, given orally exerts local and systemic anti-inflammatory effects in lipopolysaccharide-induced sepsis in mice. *Br J Pharmacol* 2009,**157**:1024-1033.

15. Kinross J, von Roon AC, Penney N, Holmes E, Silk D, Nicholson JK, Darzi A: The gut microbiota as a target for improved surgical outcome and improved patient care. *Curr Pharm Des* 2009,**15**:1537-1545.

16. Tarnow-Mordi W, Isaacs D, Dutta S: Adjunctive immunologic interventions in neonatal sepsis. *Clin Perinatol 2010*,37:481-499.

17. Awad H, Mokhtar H, Imam SS, Gad GI, Hafez H, Aboushady N: Comparison between killed and living probiotic usage versus placebo for the prevention of necrotizing enterocolitis and sepsis in neonates. *Pak J Biol Sci* 2010,**13**:253-262.

18. Forsyth CB, Farhadi A, Jakate SM, Tang Y, Shaikh M, Keshavarzian A: Lactobacillus GG treatment ameliorates alcohol-induced intestinal oxidative stress, gut leakiness, and liver injury in a rat model of alcoholic steatohepatitis. *Alcohol* 2009,**43**:163-172.

19. Cani PD, Possemiers S, Van de Wiele T, Guiot Y, Everard A, Rottier O, Geurts L, Naslain D, Neyrinck A, Lambert DM, Muccioli GG, Delzenne NM : Changes in gut microbiota control inflammation in obese mice through a mechanism involving GLP-2-driven improvement of gut permeability. *Gut* 2009,**58**:1091-1093.

20. Hsieh MH, Versalovic J: The human microbiome and probiotics: implications for pediatrics. *Curr Probl Pediatr Adolesc Health Care* 2008,**38**:309-327.

21. Madsen K: Probiotics in critically ill patients. *J Clin Gastroenterol* 2008,**42** (Suppl 3 Pt 1):S116-S118.

22. Wynn JL, Neu J, Moldawer LL, Levy O: Potential of immunomodulatory agents for prevention and treatment of neonatal sepsis. *J Perinatol* 2009,**29**:79-88.

23. Peppelenbosch MP, Ferreira CV: Immunology of pre- and probiotic supplementation*. Br J Nutr 2009*,**101**:2-4.

24. Arribas B, Rodríguez-Cabezas ME, Comalada M, Bailón E, Camuesco D, Olivares M, Xaus J, Zarzuelo A, Gálvez J: Evaluation of the preventative effects exerted by Lactobacillus fermentum in an experimental model of septic shock induced in mice*. Br J Nutr* 2009,**101**:51-58.

25. Zein EF, Karaa S, Chemaly A, Saidi I, Daou-Chahine W, Rohban R: Lactobacillus rhamnosus septicemia in a diabetic patient associated with probiotic use: a case report. *Ann Biol Clin (Paris)* 2008,**66**:195-198.

26. Fujita T: Role of probiotic bacteria in sepsis. *Hepatology* 2008,**47**:1422.

27. Matsumoto T, Ishikawa H, Tateda K, Yaeshima T, Ishibashi N, Yamaguchi K: Oral administration of Bifidobacterium longum prevents gut-derived Pseudomonas aeruginosa sepsis in mice. *J Appl Microbiol* 2008,**104**:672-680.

28. Qin X: Inactivation of digestive proteases: another mechanism that probiotics may have conferred a protection. *Am J Gastroenterol* 2007,**102**:2109.

29. Ruan X, Shi H, Xia G, Xiao Y, Dong J, Ming F, Wang S: Encapsulated Bifidobacteria reduced bacterial translocation in rats following hemorrhagic shock and resuscitation. *Nutrition* 2007,**23**:754-761.

30. Ewaschuk J, Endersby R, Thiel D, Diaz H, Backer J, Ma M, Churchill T, Madsen K: Probiotic bacteria prevent hepatic damage and maintain colonic barrier function in a mouse model of sepsis. *Hepatology* 2007**,46**:841-850.

31. Seal M, Naito Y, Barreto R, Lorenzetti A, Safran P, Marotta F: Experimental radiotherapy-induced enteritis: a probiotic interventional study. *J Dig Dis* 2007,**8**:143-147.

32. Tok D, Ilkgul O, Bengmark S, Aydede H, Erhan Y, Taneli F, Ulman C, Vatansever S, Kose C, Ok G: Pretreatment with pro- and synbiotics reduces peritonitis-induced acute lung injury in rats. *J Trauma* 2007,**62**:880-885.

33. Bell SG: Immunomodulation, Part V: Probiotics. *Neonatal Netw* 2007,**26**:57-60.

34. Bu HF, Wang X, Zhu YQ, Williams RY, Hsueh W, Zheng X, Rozenfeld RA, Zuo XL,Tan XD: Lysozyme-modified probiotic components protect rats against polymicrobial sepsis: role of macrophages and cathelicidin-related innate immunity. *J Immunol* 2006,**177**:8767-8776.

35. Hammerman C, Bin-Nun A, Kaplan M: Safety of probiotics: comparison of two popular strains. *BMJ* 2006,**333**:1006-1008.

36. Mangell P, Lennernäs P, Wang M, Olsson C, Ahrné S, Molin G, Thorlacius H, Jeppsson B: Adhesive capability of Lactobacillus plantarum 299v is important for preventing bacterial translocation in endotoxemic rats. *APMIS* 2006,**114**:611-618.

37. Saiman L: Strategies for prevention of nosocomial sepsis in the neonatal intensive care unit. *Curr Opin Pediatr* 2006,**18**:101-106.

38. Graf C, Gavazzi G: Saccharomyces cerevisiae fungemia in an immunocompromised patient not treated with Saccharomyces boulardii preparation. *J Infect* 2007,**54**:310-311.

39. Lin HC, Su BH, Chen AC, Lin TW, Tsai CH, Yeh TF, Oh W: Oral probiotics reduce the incidence and severity of necrotizing enterocolitis in very low birth weight infants. *Pediatrics* 2005,**115**:1-4.

40. Ouwehand AC, Saxelin M, Salminen S: Phenotypic differences between commercial Lactobacillus rhamnosus GG and L. rhamnosus strains recovered from blood. *Clin Infect Dis* 2004,**39**:1858-1860.

41. Young RJ, Vanderhoof JA: Two cases of Lactobacillus bacteremia during probiotic treatment of short gut syndrome*. J Pediatr Gastroenterol Nutr* 2004,**39**:436-437.

42. Jain PK, McNaught CE, Anderson AD, MacFie J, Mitchell CJ: Influence of synbiotic containing Lactobacillus acidophilus La5, Bifidobacterium lactis Bb 12, Streptococcus thermophilus, Lactobacillus bulgaricus and oligofructose on gut barrier function and sepsis in critically ill patients: a randomised controlled trial. *Clin Nutr* 2004,**23**:467-475.

43. Bengmark S: Synbiotics to strengthen gut barrier function and reduce morbidity in critically ill patients. *Clin Nutr* 2004,**23**:441-445.

44. Kunz AN, Noel JM, Fairchok MP: Two cases of Lactobacillus bacteremia during probiotic treatment of short gut syndrome. *J Pediatr Gastroenterol Nutr* 2004,**38**:457-458.

45. Salminen MK, Rautelin H, Tynkkynen S: Lactobacillus bacteremia, clinical significance, and patient outcome, with special focus on probiotic L. rhamnosus GG. *Clin Infect Dis* 2004,**38**:62-69.

46. Lestin F, Pertschy A, Rimek D: Fungemia after oral treatment with Saccharomyces boulardii in a patient with multiple comorbidities. *Dtsch Med Wochenschr* 2003,**128**:2531-2533.

47. Kecskés G, Belágyi T, Oláh A: Early jejunal nutrition with combined pre- and probiotics in acute pancreatitis--prospective, randomized, double-blind investigations] *Magy Seb* 2003,**56**:3-8.

48. Jirapinyo P, Densupsoontorn N, Thamonsiri N, Wongarn R: Prevention of antibiotic-associated diarrhea in infants by probiotics. *J Med Assoc Thai* 2002,**85** (Suppl 2):S739-S742.

49. Chiva M, Soriano G, Rochat I, Peralta C, Rochat F, Llovet T, Mirelis B, Schiffrin EJ, Guarner C, Balanzó J: Effect of Lactobacillus johnsonii La1 and antioxidants on intestinal flora and bacterial translocation in rats with experimental cirrhosis*. J Hepatol* 2002,**37**:456-462.

50. Periti P, Tonelli F: Preclinical and clinical pharmacology of biotherapeutic agents: Saccharomyces boulardii. *J Chemother* 2001,**13**:473-493.

51. Apostolou E, Kirjavainen PV, Saxelin M, Rautelin H, Valtonen V, Salminen SJ, Ouwehand AC: Good adhesion properties of probiotics: a potential risk for bacteremia? *FEMS Immunol Med Microbiol* 2001,**31**:35-39.

52.Niault M, Thomas F, Prost J, Ansari FH, Kalfon P: Fungemia due to Saccharomyces species in a patient treated with enteral Saccharomyces boulardii. *Clin Infect Dis* 1999,**28**:930.

**5) "Probiotics"[Mesh] AND ("Informed Consent"[Mesh] OR "Consent Forms"[Mesh])**

1. Sharp RR, Achkar JP, Brinich MA, Farrell RM: Helping patients make informed choices about probiotics: a need for research. *Am J Gastroenterol* 2009,**104**:809-813.

**6) "Probiotics"[Mesh] AND "Legislation, Drug"[Mesh]**

1. Ross JJ, Boucher PE, Bhattacharyya SP, Kopecko DJ, Sutkowski EM, Rohan PJ,

Chandler DK, Vaillancourt J: Considerations in the development of live biotherapeutic products for clinical use. *Curr Issues Mol Biol* 2008,**10**:13-16.

2. Hoffman FA: Business considerations in the development of probiotics. *Clin Infect Dis* 2008,**46** (Suppl 2):S141-S143.

3. Hibberd PL, Davidson L: Probiotic foods and drugs: impact of US regulatory status on design of clinical trials. *Clin Infect Dis* 2008,**46** (Suppl 2):S137-S140.

4. Degnan FH: The US Food and Drug Administration and probiotics: regulatory categorization. *Clin Infect Dis* 2008,**46** (Suppl 2):S133-S136.

5. Hoffman FA: Development of probiotics as biologic drugs. *Clin Infect Dis* 2008, **46** (Suppl 2):S125-S127.

6. Mattia A, Merker R: Regulation of probiotic substances as ingredients in foods: premarket approval or "generally recognized as safe" notification*. Clin Infect Dis* 2008,**46** (Suppl 2):S115-S118.

7. Sutton A: Environmental assessment requirements for live biological drugs. *Clin Infect Dis* 2008,**46** (Suppl 2):S112-S114.

8. Khan SH, Ansari FA: Probiotics--the friendly bacteria with market potential in global market. *Pak J Pharm Sci* 2007,**20**:76-82.

9. Henriksson A, Borody T, Clancy R: Probiotics under the regulatory microscope. *Expert Opin Drug Saf* 2005,**4**:1135-1143.

10. Halsted CH: Dietary supplements and functional foods: 2 sides of a coin? *Am J Clin Nutr* 2003,**77**(Suppl 4):1001S-1007S.

**7) "Probiotics"[Mesh] AND ("Ethics"[Mesh] OR "Ethics Committees"[Mesh] OR "Ethics Committees, Clinical"[Mesh] OR "Codes of Ethics"[Mesh] OR "Ethics Committees, Research"[Mesh] OR "Ethics, Clinical"[Mesh] OR "Ethics, Professional"[Mesh] OR "Ethics, Medical"[Mesh] OR "Bioethics"[Mesh])**

1. Neu J, Shuster J: Nonadministration of routine probiotics unethical--really? *Pediatrics* 2010,**126**:e740-e741.

2. Tijssen JG: PROPATRIA and safety in clinical trials. Comments on the IGZ-CCMO-VWA-raport. *Ned Tijdschr Geneeskd* 2009,**153**:B520.

3. van Maanen H: Rash invocation of the Ingelfinger rule hinders scientific communication*. Ned Tijdschr Geneeskd* 2008,**152**:666-667.

**8) "Probiotics"[Mesh] AND Refrigeration"[Mesh]**

1. Liu SQ, Tsao M: Enhancement of survival of probiotic and non-probiotic lactic acid bacteria by yeasts in fermented milk under non-refrigerated conditions. *Int J Food Microbiol* 2009,**135**:34-38.

2. Goderska K, Czarnecki Z: Influence of microencapsulation and spray drying on the viability of Lactobacillus and Bifidobacterium strains. *Pol J Microbiol* 2008,**57**:135-140.

3. Tapia MS, Rojas-Graü MA, Rodríguez FJ, Ramírez J, Carmona A, Martin-Belloso O: Alginate- and gellan-based edible films for probiotic coatings on fresh-cut fruits. *J Food Sci* 2007,**72**:E190-E196.

4. Patrignani F, Iucci L, Lanciotti R, Vallicelli M, Mathara JM, Holzapfel WH, Guerzoni ME: Effect of high-pressure homogenization, nonfat milk solids, and milkfat on the technological performance of a functional strain for the production of probiotic fermented milks. *J Dairy Sci* 2007,**90**:4513-4523.

5. Angelov A, Gotcheva V, Kuncheva R, Hristozova T: Development of a new oat-based probiotic drink. *Int J Food Microbiol* 2006,**112**:75-80.

6. Kongo JM, Gomes AM, Malcata FX: Manufacturing of fermented goat milk with a mixed starter culture of Bifidobacterium animalis and Lactobacillus acidophilus in a controlled bioreactor. *Lett Appl Microbiol* 2006,**42**:595-599.

7. Guetmonde M, Nieves C, Vinderola G, Reinheimer J, de los Reyes-Gavilan CG: Evolution of carbohydrate fraction in carbonated fermented milks as affected by

beta-galactosidase activity of starter strains. *J Dairy Res* 2002,**69**:125-137.

8. Adhikari K, Mustapha A, Grün IU, Fernando L: Viability of microencapsulated bifidobacteria in set yogurt during refrigerated storage*. J Dairy Sci* 2000,**83**:1946-1951.

9. Schillinger U: Isolation and identification of lactobacilli from novel-type probiotic and mild yoghurts and their stability during refrigerated storage. *Int J Food Microbiol* 1999,**47**:79-87.

**9) "Probiotics"[Mesh] AND "Quality Control"[Mesh]**

1. Drago L, Rodighiero V, Celeste T, Rovetto L, DE Vecchi E: Microbiological evaluation of commercial probiotic products available in the USA in 2009. *J Chemother* 2011, **22**:373-377.

2. Tuomola E, Crittenden R, Playne M, Isolauri E, Salminen S: [Quality assurance criteria for probiotic bacteria.](http://www.ncbi.nlm.nih.gov/pubmed/11157347) *Am J Clin Nutr* 2001,**73**(2 Suppl):393S-398S

**10) "Probiotics"[Mesh] AND "Quality Assurance, Health Care"[Mesh]**

No relevant articles.
