# Supplementary material for: Evidence-based guidelines for use of probiotics in preterm neonates
Source: BMC Med. 2011 Aug 2;9:92. doi: 10.1186/1741-7015-9-92 (PMC3163616; doi:10.1186/1741-7015-9-92)
Supplement: Additional file 2 — Appendix I - EMBASE search results. This appendix includes the results of the EMBASE (1980 to October 2010) search. [file 1741-7015-9-92-S2.DOC]

**Appendix II: Embase search results**

| **MeSH word** | **Yield from initial search** | **Relevant articles** |
| --- | --- | --- |
| 1) probiotic.mp. or probiotic agent AND microbiological examination/ or culture medium/ or methodology/ or culture methods.mp. or culture technique/ or bacterium culture/ | 1074 | 17 |
| 2) probiotic.mp. or probiotic agent AND antibiotic susceptibility.mp. or antibiotic sensitivity | 153 | 24 |
| 3) probiotic.mp. or probiotic agent AND newborn sepsis/ or gram negative sepsis/ or sepsis/ | 225 | 12 |
| 4) probiotic.mp. or probiotic agent AND bacterial translocation | 205 | 33 |
| 5) probiotic.mp. or probiotic agent AND legistlation.mp. or licence/ or law/ | 46 | 9 |
| 6) probiotic.mp. or probiotic agent AND medical ethics/ or research ethics/ or professional standard/ | 14 | 14 |
| 7) probiotic.mp. or probiotic agent AND  informed consent | 14 | 0 |
| 8) probiotics.mp. or probiotic agent AND temperature/ or drug storage/ or drug packaging/ or cold chain.mp. or drug stability/ or freezing/ | 135 | 37 |
| 9) probiotic.mp. or probiotic agent quality assurance.mp. or quality control/ | 77 | 4 |

**Embase search strategy details including references:**

1) **probiotic.mp. or probiotic agent AND microbiological examination/ or culture medium/ or methodology/ or culture methods.mp. or culture technique/ or bacterium culture/**

1. Mileti E, Matteoli G, Iliev ID, Rescigno M: Comparison of the immunomodulatory properties of three probiotic strains of Lactobacilli using complex culture systems: Prediction for in vivo efficacy*. PLoS ONE* 2009, **4**: e7056.

2. Kramer M, Obermajer N, Bogovic Matijasic B, Rogelj I, Kmetec V: Quantification of live and dead probiotic bacteria in lyophilised product by real-time PCR and by flow cytometry. *Applied Microbiology and Biotechnology* 2009, **84:** 1137-1147.

3. Siaterlis A, Deepika G, Charalampopoulos D: Effect of culture medium and cryoprotectants on the growth and survival of probiotic lactobacilli during freeze drying. *Letters in Applied Microbiology* 2009, **48:** 295-301.

4. Ren YF, Wang L: Effects of probiotics on intestinal bacterial colonization in premature infants. *Chinese Journal of Contemporary Pediatrics* 2010, **12**:192-194.

5. Rada V, Splichal I, Rockova S, Grmanova M, Vlkova E: Susceptibility of bifidobacteria to lysozyme as a possible selection criterion for probiotic bifidobacterial strains. *Biotechnology Letters* 2010, **32:** 451-455.

6. Abdulamir AS, Yoke TS, Nordin N, Abu Bakar F: Detection and quantification of probiotic bacteria using optimized DNA extraction, traditional and real-time PCR methods in complex microbial communities. *African Journal of Biotechnology* 2010**, 9**:1481-1492.

7. Liu C, Zhang ZY, Dong K, Yuan JP, Guo XK: Antibiotic resistance of probiotic strains of lactic acid bacteria isolated from marketed foods and drugs. *Biomedical and Environmental Sciences* 2009, **22:** 401-412.

8 Sleator RD, Hill C: Rational design of improved pharmabiotics. *Journal of biomedicine & biotechnology* 2009, Published online Sept 10 (Article ID: 275287):1-7.

9. Minocha A: Probiotics for preventive health. Nutrition in clinical practice: official publication of the *American Society for Parenteral and Enteral Nutrition* 2009, **24**: 227-241.

10. Meadows-Oliver M, Reid V: Use of Probiotics in Pediatrics*. Journal of Pediatric Health Care* 2009, **23**:194-197.

13. Ding WK, Shah NP: An improved method of microencapsulation of probiotic bacteria for their stability in acidic and bile conditions during storage. *Journal of Food Science* 2009, **74**: 53-61.

14. Koretz RL: Probiotics, critical illness, and methodologic bias. Nutrition in clinical practice: official publication of the *American Society for Parenteral and Enteral Nutrition* 2009, **24**:45-49.

15. Koskenniemi K, Koponen J, Kankainen M, Savijoki K, Tynkkynen S, De Vos WM, Kalkkinen N, Varmanen P: Proteome analysis of Lactobacillus rhamnosus GG using 2-D DIGE and mass spectrometry shows differential protein production in laboratory and industrial-type growth media. *Journal of Proteome Research* 2009, **8**: 4993-5007.

16. Klayraung S, Viernstein H, Okonogi S: Development of tablets containing probiotics: Effects of formulation and processing parameters on bacterial viability. *International Journal of Pharmaceutics* 2009, **370**:54-60.

17. Saulnier DM, Kolida S, Gibson GR: Microbiology of the human intestinal tract and approaches for its dietary modulation. *Current Pharmaceutical Design* 2009, **15**:1403-1414.

**2) probiotic.mp. or probiotic agent AND antibiotic susceptibility.mp. or antibiotic sensitivity**

1. Xiao JZ, Takahashi S, Odamaki T, Yaeshima T, Iwatsuki K: Antibiotic susceptibility of bifidobacterial strains distributed in the Japanese market. *Bioscience, Biotechnology and Biochemistry* 2010, **74**:336-342.

2. Hammad A, Shimamoto T: Antibiotic resistance of the Japanese probiotic bacteria. International Journal of Antimicrobial Agents. Conference: 26th International Congress of Chemotherapy and Infection Toronto, ON Canada 2009.

3. Reyed M: Antibiogram: profile of potential probiotic Bifidobacterium spp. recovered from faeces sample of human origin. *Clinical Microbiology and Infection*. Conference: 19th European Congress of Clinical Microbiology and Infectious Diseases (ECCMID) Helsinki Finland. Conference Publication: (var.pagings) 2009, 15: S624-S625.

4. Verdenelli MC, Ghelfi F, Silvi S, Orpianesi C, Cecchini C, Cresci A: Probiotic properties of Lactobacillus rhamnosus and Lactobacillus paracasei isolated from human faeces*. European Journal of Nutrition* 2009, **48**:355-363.

5. Cordoba M, Chaves C, Arias ML: Identification, quantification and antimicrobial susceptibility pattern of probiotic bacteria added to common use food products in Costa Rica. *Archivos Latinoamericanos de Nutricion* 2009, **59**:179-183.

6. Xu J, Liu X, Yang B, Li Z: Antimicrobial susceptibility of probiotics. Wei sheng yan jiu- *Journal of hygiene research* 2008, **37**:354-356.

7. Hammad AM, Shimamoto T: Towards a compatible probiotic-antibiotic combination therapy: Assessment of antimicrobial resistance in the Japanese probiotics. *Journal of Applied Microbiology* 2010, **109**:1349-1360.

8. Danielsen M, Seifert J: The development of an international ISO/IDF standard for susceptibility testing of lactic acid bacteria and bifidobacteria based on contributions from prosafe and ACE-ART. *International Journal of Probiotics and Prebiotics* 2008, **3**: 247-248.

9. Pawan R, Bhatia A: Isolation, identification, biochemical characterization and probiotics functionality of faecal lab isolates. *International Journal of Probiotics and Prebiotics* 2008, **3**:191-198.

10. Blandino G., Milazzo I, Fazio D: Antibiotic susceptibility of bacterial isolates from probiotic products available in Italy*. Microbial Ecology in Health and Disease* 2008, **20**:199-203.

11. Sutton A: Product development of probiotics as biological drugs. *Clinical Infectious Diseases* 2008, **46** (Suppl 2):S128-S132.

12. Snydman DR: The safety of probiotics. *Clinical Infectious Diseases* 2008,**46** (Suppl 2): S104-S111.

13. Klare I, Konstabel C, Werner G, Huys G, Vankerckhoven V, Kahlmeter G, Hildebrandt B, Müller-Bertling S, Witte W, Goossens H: Antimicrobial susceptibilities of Lactobacillus, Pediococcus and Lactococcus human isolates and cultures intended for probiotic or nutritional use. *Journal of Antimicrobial Chemotherapy* 2007, **59**: 900-912.

14. Milazzo I, Speciale A, Musumeci R, Fazio D, Blandino G: Identification and antibiotic susceptibility of bacterial isolates from probiotic products available in Italy.  *New Microbiologica* 2006, **29**:281-291.

15. Masco L, Van Hoorde K, De Brandt E, Swings J, Huys G: Antimicrobial susceptibility of Bifidobacterium strains from humans, animals and probiotic products. *Journal of Antimicrobial Chemotherapy* 2006, **58**: 85-94.

16. Land MH, [Rouster-Stevens K](http://www.ncbi.nlm.nih.gov/pubmed?term="Rouster-Stevens K"%5BAuthor%5D), [Woods CR](http://www.ncbi.nlm.nih.gov/pubmed?term="Woods CR"%5BAuthor%5D), [Cannon ML](http://www.ncbi.nlm.nih.gov/pubmed?term="Cannon ML"%5BAuthor%5D), [Cnota J](http://www.ncbi.nlm.nih.gov/pubmed?term="Cnota J"%5BAuthor%5D), [Shetty AK](http://www.ncbi.nlm.nih.gov/pubmed?term="Shetty AK"%5BAuthor%5D): Lactobacillus sepsis associated with probiotic therapy. *Pediatrics* 2005, **115**:178-181.

17. Fernandez MF, Boris S, Barbes C: Safety evaluation of Lactobacillus delbrueckii subsp. lactis UO 004, a probiotic bacterium. *Research in Microbiology* 2005, **156:**154-160.

18. Moubareck C, Gavini F, Vaugien L, Butel MJ, Doucet-Populaire F: Antimicrobial susceptibility of bifidobacteria. *Journal of Antimicrobial Chemotherapy* 2005, **55**: 38-44.

19. Ventura M, van Sinderen D, Fitzgerald GF, Zink R: Insights into the taxonomy, genetics and physiology of bifidobacteria. Antonie van Leeuwenhoek, *International Journal of General and Molecular Microbiology* 2004, **86**: 205-223.

20. Chou YK, Chen HJ, Shaio MF, Kuo YM: In vitro antibiotic susceptibility of Lactobacilli isolated from commercial products containing active Lactobacilli. *Acta Paediatrica Taiwanica* 2004, **45**:141-144.

21. Temmerman R, Pot B, Huys G, Swings J: Identification and antibiotic susceptibility of bacterial isolates from probiotic products. *International Journal of Food Microbiology* 2003, **81**:1-10.

22. Saarela M, Matto J, Mattila-Sandholm T: Safety aspects of Lactobacillus and Bifidobacterium species originating from human oro-gastrointestinal tract or from probiotic products. *Microbial Ecology in Health and Disease* 2002, **14**:233-240.

23. harteris WP, Kelly PM, Morelli L, Collins JK: Gradient diffusion antibiotic susceptibility testing of potentially probiotic lactobacilli. *Journal of Food Protection* 2001, **64**:2007-2014.

24. Charteris WP, Kelly PM, Morelli L, Collins JK: Antibiotic susceptibility of potentially probiotic Lactobacillus species. *Journal of Food Protection* 1998, 61: 1636-1643.

**3) probiotic.mp. or probiotic agent AND newborn sepsis/ or gram negative sepsis/ or sepsis/**

1. Zein EF, Karaa S, Chemaly A, Saidi I, Daou-Chahine W, Rohban R: Lactobacillus rhamnosus septicemia in a diabetic patient associated with probiotic use: A case report. *Annales de Biologie Clinique*. 2008, **66**:195-198.

2. . Hammerman C, Bin-Nun A, Kaplan M: Safety of probiotics: Comparison of two popular strains. *British Medical Journal* 2006, **333**:1006-1008.

3. Boyle RJ, Robins-Browne RM, Tang MLK: Probiotic use in clinical practice: What are the risks? *American Journal of Clinical Nutrition* 2006, **83**:1256-1264.

4. Kunz AN, Fairchok MP, Noel JM: Lactobacillus sepsis associated with probiotic therapy. *Pediatrics* 2005, **116**:517.

5. Berger RE: Lactobacillus sepsis associated with probiotic therapy. *The Journal of Urology* 2005; **174**:1843.

6. Land MH, Rouster-Stevens K, Woods CR, Cannon ML, Cnota J, Shetty AK: Lactobacillus sepsis associated with probiotic therapy. *Pediatrics* 2005, **115**:178-181.

7. Benchimol EI, Mack DR: Safety issues of probiotic ingestion. *Practical Gastroenterology* 2005, **29**: 23-34.

8. Manzoni P, Lista G, Gallo E: Routinary probiotic Lactobacillus rhamnosus GG administration in preterm very-low-birth-weight neonates: A retrospective, 6-year cohort study from two large tertiary NICUs in Italy. *Pediatric Research,* Conference: 50th Annual Midwest Society for Pediatric Research - Scientific Meeting Chicago, IL United States 2009, **66:** S94.

9. Awad H, Mokhtar G, Imam SS: Comparison between killed and living probiotic usage versus placebo for the prevention of necrotizing enterocolitis and sepsis in neonates. *Pakistan Journal of Biological Sciences* 2010, **13**:253-262.

10. Kataria J, Li N, Wynn JL, Neu J: Probiotic microbes: Do they need to be alive to be beneficial? Nutrition Reviews 2009, **67**:546-550.

11. Besselink MG, van Santvoort HC, Buskens E, Boermeester MA, van Goor H, Timmerman HM, Nieuwenhuijs VB, Bollen TL, van Ramshorst B, Witteman BJ, Rosman C, Ploeg RJ, Brink MA, Schaapherder AF, Dejong CH, Wahab PJ, van Laarhoven CJ, van der Harst E, van Eijck CH, Cuesta MA, Akkermans LM, Gooszen HG; Acute Pancreatitis Werkgroep Nederland. Probiotic prophylaxis in patients with predicted severe acute pancreatitis: A randomised, double-blind, placebo-controlled trial. *Nederlands Tijdschrift voor Geneeskunde* 2008, **152**:685-696.

12. Alberda C, [Gramlich L](http://www.ncbi.nlm.nih.gov/pubmed?term="Gramlich L"%5BAuthor%5D), [Meddings J](http://www.ncbi.nlm.nih.gov/pubmed?term="Meddings J"%5BAuthor%5D), [Field C](http://www.ncbi.nlm.nih.gov/pubmed?term="Field C"%5BAuthor%5D), [McCargar L](http://www.ncbi.nlm.nih.gov/pubmed?term="McCargar L"%5BAuthor%5D), [Kutsogiannis D](http://www.ncbi.nlm.nih.gov/pubmed?term="Kutsogiannis D"%5BAuthor%5D), [Fedorak R](http://www.ncbi.nlm.nih.gov/pubmed?term="Fedorak R"%5BAuthor%5D), [Madsen K](http://www.ncbi.nlm.nih.gov/pubmed?term="Madsen K"%5BAuthor%5D): Effects of probiotic therapy in critically ill patients: A randomized, double-blind, placebo-controlled trial. *American Journal of Clinical Nutrition* 2007, **85**:816-823.

**4) probiotic.mp. or probiotic agent AND bacterial translocation**

1. Wang JQ, Ding ZX, Zhang M: Effect of probiotics on intestinal flora disturbance and bacterial translocation in mice with spontaneous colitis*. Journal of Shanghai Jiaotong University (Medical Science)* 2010, **30**:186-190.

2. Abe F, Muto M, Yaeshima T, Iwatsuki K, Aihara H, Ohashi Y, Fujisawa T: Safety evaluation of probiotic bifidobacteria by analysis of mucin degradation activity and translocation ability. *Anaerobe* 2010, **16**:131-136.

3. Lata J. Juránková J, Stibůrek O, Príbramská V, Senkyrík M, Vanásek T: Probiotics in acute pancreatitis - A randomised, placebo-controlled, double-blind study. Vnitr Lek 2010, **56**:111-114.

4. Cukrowska B, Motyl I, Kozáková H, Schwarzer M, Górecki RK, Klewicka E, Slizewska K, Libudzisz Z: Probiotic Lactobacillus strains: in vitro and in vivo studies. *Folia Microbiol* 2009, **54**:533-537.

5. Whelan K, Myers CE: Safety of probiotics in patients receiving nutritional support: A systematic review of case reports, randomized controlled trials, and nonrandomized trials. *American Journal of Clinical Nutrition* 2010, **91**:687-703.

6. Besselink MG, van Santvoort HC, Renooij W, de Smet MB, Boermeester MA, Fischer K, Timmerman HM, Ahmed Ali U, Cirkel GA, Bollen TL, van Ramshorst B, Schaapherder AF, Witteman BJ, Ploeg RJ, van Goor H, van Laarhoven CJ, Tan AC, Brink MA, van der Harst E, Wahab PJ, van Eijck CH, Dejong CH, van Erpecum KJ, Akkermans LM, Gooszen HG; Dutch Acute Pancreatitis Study Group: Intestinal barrier dysfunction in a randomized trial of a specific probiotic composition in acute pancreatitis. *Annals of Surgery* 2009, **250**: 712-719.

7. Copeland DR, McVay MR, Dassinger MS, Jackson RJ, Smith SD: Probiotic fortified diet reduces bacterial colonization and translocation in a long-term neonatal rabbit model. *Journal of Pediatric Surgery* 2009, **44**: 1061-1064.

8. Yakabe T, Moore EL, Yokota S, Sui H, Nobuta Y, Fukao M, Palmer H, Yajima N: Safety assessment of Lactobacillus brevis KB290 as a probiotic strain. *Food and Chemical Toxicology* 2009, **47**: 2450-2453.

9. Rayes N, Seehofer D, Neuhaus P: Prebiotics, probiotics, synbiotics in surgery-Are they only trendy, truly effective or even dangerous? *Langenbeck's Archives of Surgery* 2009, **394**:547-555.

10. Sherman MP: New Concepts of Microbial Translocation in the Neonatal Intestine: Mechanisms and Prevention. *Clinics in Perinatology* 2010, **37**:565-579.

11. Garcia-Urkia N, Aldazabal P, Asensio AB, García-Arenzana JM, Bachiller P, Eizaguirre I: Short bowel syndrome in the research setting: 15 years' experience. *Cirugia pediatrica : organo oficial de la Sociedad Espanola de Cirugia Pediatrica* 2008, **21**:55-61.

12. Soeters PB: Probiotics: Did we go wrong, and if so, where? *Clinical Nutrition* 2010, **27**:173-178.

13. Kabeir BM, Yazid AM, Stephenie W, Nazrul Hakim M, Muhammad Anas O, Shuhaimi M: Safety evaluation of Bifidobacterium pseudocatenulatum G4 as assessed in BALB/c mice. *Letters in Applied Microbiology* 2008, **46**: 32-37.

14. McVay MR, Boneti C, Habib CM, Keller JE, Kokoska ER, Jackson RJ, Smith SD: Formula fortified with live probiotic culture reduces pulmonary and gastrointestinal bacterial colonization and translocation in a newborn animal model. *Journal of Pediatric Surgery* 2008**, 43**: 25-29.

15. Lara-Villoslada F, Sierra S, Diaz-Ropero MP, Olivares M, Xaus J: Safety assessment of the human milk-isolated probiotic Lactobacillus salivarius CECT5713. *Journal of Dairy Science* 2007, **90**:3583-3589.

16. Lara-Villoslada F, Sierra S, Martin R, Delgado S, Rodriguez JM, Olivares M, Xaus J: Safety assessment of two probiotic strains, Lactobacillus coryniformis CECT5711 and Lactobacillus gasseri CECT5714. *Journal of Applied Microbiology* 2007, **103**:175-184.

17. Zareie M, Johnson-Henry K, Jury J, Yang PC, Ngan BY, McKay DM, Soderholm JD, Perdue MH, Sherman PM: Probiotics prevent bacterial translocation and improve intestinal barrier function in rats following chronic psychological stress. *Gut* 2006**, 55**:1553-1560.

18. Ljungh A, Wadstrom T: Lactic acid bacteria as probiotics. *Current Issues in Intestinal Microbiology* 2006, **7**: 73-90.

19. Gun F, Salman T, Gurler N, Olgac V: Effect of probiotic supplementation on bacterial translocation in thermal injury. Surgery Today 2005, **35**: 760-764.

20. [Lee YH](http://agris.fao.org/?query=%2Bauthor:"Lee, Y.H."), [Moon EP,](http://agris.fao.org/?query=%2Bauthor:"Moon, E.P.") [Seok SH,](http://agris.fao.org/?query=%2Bauthor:"Seok, S.H.") [Baek MW,](http://agris.fao.org/?query=%2Bauthor:"Baek, M.W.") [Lee HY,](http://agris.fao.org/?query=%2Bauthor:"Lee, H.Y.") [Kim DJ,](http://agris.fao.org/?query=%2Bauthor:"Kim, D.J.") [Kim CH](http://agris.fao.org/?query=%2Bauthor:"Kim, C.H."), [Park JH](http://agris.fao.org/?query=%2Bauthor:"Park, J.H."): Safety assessment of Lactobacillus fermentum PL9005, a potential probiotic lactic acid bacterium, in mice. *Journal of Microbiology and Biotechnology* 2005, **15**: 603-608.

21. Luyer MD, Buurman WA, Hadfoune M, Speelmans G, Knol J, Jacobs JA, Dejong CH, Vriesema AJ, Greve JW: Strain-specific effects of probiotics on gut barrier integrity following hemorrhagic shock. *Infection and Immunity* 2005, **73**: 3686-3692.

22. Qin HL, Shen TY, Gao ZG, Fan XB, Hang XM, Jiang YQ, Zhang HZ: Effect of lactobacillus on the gut microflora and barrier function of the rats with abdominal infection. *World Journal of Gastroenterology* 2005; **11**:2591-2596.

23. Osman N, Adawi D. Ahrne S, Jeppsson B, Molin G: Probiotic strains of Lactobacillus and Bifidobacterium affect the translocation and intestinal load of Enterobacteriaceae differently after D-galactosamine-induced liver injury in rats. *Microbial Ecology in Health and Disease* 2005, **17**:40-46.

24. Jeppsson B, Mangell P, Adawi D, Molin G: Bacterial translocation: Impact of probiotics. *Scandinavian Journal of Nutrition/Naringsforskning* 2004, **48**: 37-41.

25. Seehofer D, Rayes N, Schiller R, Stockmann M, Müller AR, Schirmeier A, Schaeper F, Tullius SG, Bengmark S, Neuhaus P: Probiotics partly reverse increased bacterial translocation after simultaneous liver resection and colonic anastomosis in rats. *Journal of Surgical Research* 2004, **117**:262-271.

26. Marteau P, Shanahan F: Basic aspects and pharmacology of probiotics: An overview of pharmacokinetics, mechanisms of action and side-effects. *Bailliere's Best Practice and Research in Clinical Gastroenterology* 2003, **17**:725-740.

27. Jeppsson B, Mangell P, Adawi D, Molin G: Bacterial translocation: Impact of luminal content on barrier function. *Archives of Hellenic Medicine* 2003, **20** (Suppl A): 68-72.

28. Garcia-Urkia N, Asensio AB, Zubillaga Azpiroz I, Zubillaga Huici P, Vidales C, García-Arenzana JM, Aldazábal P, Eizaguirre I: Beneficial effects of Bifidobacterium lactis in the prevention of bacterial translocation in experimental short bowel syndrome. *Cirugia pediatrica : organo oficial de la Sociedad Espanola de Cirugia Pediatrica* 2002, **15**: 162-165.

29. Eizaguirre I, Urkia NG, Asensio AB, Zubillaga I, Zubillaga P, Vidales C, Garcia-Arenzana JM, Aldazabal P: Probiotic supplementation reduces the risk of bacterial translocation in experimental short bowel syndrome. *Journal of Pediatric Surgery* 2002, **37**:699-702.

30. Mullie C, Romond MB, Yazourh A, Behra-Miellet J, Bezirtzoglou E, Romond R: Modulation of bacterial translocation in mice mediated through viable Bifidobacterium breve or cell-free whey intake. *Microbial Ecology in Health and Disease* 2001, **13**:160-165.

31. Mattar AF, Drongowski RA, Coran AG, Harmon CM: Effect of probiotics on enterocyte bacterial translocation in vitro. *Pediatric Surgery International* 2001, **17**: 265-268.

32. Mangiante G, Canepari P, Colucci G, Marinello P, Signoretto C, Nicoli N, Bengmark S: A probiotic as an antagonist of bacterial translocation in experimental pancreatitis. *Chirurgia Italiana* 1999, **51**:221-226.

33. Urao M, Fujimoto T, Lane GJ, Seo GI, Miyano T: Does probiotics administration decrease serum endotoxin levels in infants? *Journal of Pediatric Surgery* 1999, **34**: 273-276.

**5) probiotic.mp. or probiotic agent AND legistlation.mp. or licence/ or law/**

1. Martin E: European food watchdog slashes dubious health claims. *Science* 2010, **327**:1189.

2. Vitoria Minana I, Dalmau Serra J: Functional food in pediatrics, current legal situation and practical implications. (In Spanish) *Acta Pediatr Esp* 2009, **67**:223-230.

3. Vogel L: European probiotics industry fears regulations will scuttle market for health-promoting or disease-preventing foods. *CMAJ* 2010, **182**:E493-E494.

4. Wassenaar TM, Klein G: Safety aspects and implications of regulation of probiotic bacteria in food and food supplements. *J Food Protection* 2008, **71:**1734-1741.

5. Degnan FH: The US food and drug administration and probiotics: Regulatory categorization. *Clin Infect. Dis* 2008, **46**(Suppl 2):S133-S136.

6. Sutton A: Product development of probiotics as biological drugs. *Clin Infect Dis* 2008, **46**(Suppl 2):S128-S132.

7. Hoffman FA: Development of probiotics as biologic drugs. *Clin Infect Dis* 2008, **46**:S125-S127.

8. Saldanha LG: US food and drug administration regulations governing label claims for food products, including probiotics. *Clin Infect Dis* 2008, **46**(Suppl 2):S119-S121.

9. Mattia A, Merker R: Regulation of probiotic substances as ingredients in foods: Premarket approval or "generally recognized as safe" notification. *Clin Infect Dis* 2008, **46**(Suppl 2):S115-S118.

10. Khan SH, Ansari FA: Probiotics--the friendly bacteria with market potential in global market. *Pak J Pharm Sci* 2007, **20:**76-82.

11. Arvanitoyannis IS, Van Houwelingen-Koukaliaroglou M: Functional foods: a survey of health claims, pros and cons, and current legislation. *Crit Rev Food Sci Nutr* 2005, **45**:385-404.

12. Mack DR: Probiotics: Mixed messages. *Can Fam Phys* 2005, **51**:1455-1457+1462-1464.

13. van Niekerk JP deV: Probiotics need legislation. *S Afr Med J* 2005, **95**:70.

14. von Wright A: Regulating the safety of probiotics - The European approach. *Curr Pharm Des* 2005, **11**:17-23.

15. Prevot MB: New regulatory trends for probiotics. *J Clin Gastroenterol* 2004, **38**(Suppl 6):S61-63.

16. Drago L, De Vecchi E, Nicola L, Colombo A, Gismondo MR: Microbiological evaluation of commercial probiotic products available in Italy. *J Chemother* 2004, **16**:463-467.

17. Szajewska H, Fordymacka A, Bardowski J, Górecki RK, Mrukowicz JZ, Banaszkiewicz A: Microbiological and genetic analysis of probiotic products licensed for medicinal purposes. *Med Sci Monit* 2004, **10**:BR346-BR350.

18. Becquet P: EU assessment of enterococci as feed additives*. Int J Food Microbiol* 2003, **88**:247-254.

19. No authors listed: To ban or not to ban? *Lancet Infect Dis* 2003, 3:1.

20. McKevith B: EU legislation for health claims on the way. *Nutr Bull* 2002, **27**:185-186.

21. Przyrembel H: Consideration of possible legislation within existing regulatory frameworks. *Am J Clin Nutr* 2001, **73**(Suppl 2):471S-475S.

**6) probiotic.mp. or probiotic agent AND medical ethics/ or research ethics/ or professional standard/**

1. Gill RD: Statistical safety valve did not work for Propatria: Being unblind is sometimes necessary. *Phar Weekbl* 2010, **145**:25.

2. Gooszen HG: The PROPATRIA trial: best practices at the time were followed. *Lancet* 2010, **375**:1249-1250.

3. Besselink MGH, van der Graaf Y, Gooszen HG: Interim analysis in randomized trials: DAMOCLES' sword? *J Clin Epidemiol* 2010, **63**:353-354.

4. Giard RWM: A probiotics trial on trial: the problem of timely detection of adverse advents in therapeutic trials. *J Clin Epidemiol* 2010, **63**:347-349.

5. Tijssen JG: PROPATRIA and safety in clinical trials. Comments on the IGZ-CCMO-VWA-raport. *Ned Tijdschr Geneeskd* 2009, **153**: B520.

6. Sheldon T: Dutch probiotics study is criticised for its "design, approval, and conduct". *BMJ* 2010, **340**:70.

7. Wilkinson D. Therapeutic hypothermia and the `equal air-time' solution for controversial randomised trials. Journal of Paediatrics and Child Health 2010, 46: 577-578.

8. Coccorullo P, Strisciuglio C, Martinelli M, Miele E, Greco L, Staiano A: Lactobacillus reuteri (DSM 17938) in infants with functional chronic constipation: a double-blind, randomized, placebo-controlled study. J Pediatr 2010, **15**7:598-602. Epub 2010 Jun 12.

9. Engler RJM, With CM, Gregory PJ, Jellin JM: Complementary and alternative medicine for the allergist-immunologist: Where do I start? *J Allergy Clin Immunol* 2009, **123**:309-316.e4.

10. Neu J, Shuster J: Nonadministration of routine probiotics unethical - Really? Pediatrics 2010, **126**:e740-e741.

11. Tarnow-Mordi WO, Wilkinson D, Trivedi A, Brok J: Probiotics reduce all-cause mortality and necrotizing enterocolitis: it is time to change practice. Pediatrics 2010, 126:e743-e744.

12. Vohra S, Kemper KJ, Walls R: The use of complementary and alternative medicine in pediatrics*. Pediatrics* 2008, **122:**1374-1386.

13. Eichenwald EC, Stark AR: Management and outcomes of very low birth weight. *New Engl J Med* 2008, **358**:1700-1711+1662.

14. Verbeken G, De Vos D, Vaneechoutte M, Merabishvili M, Zizi M, Pirnay JP: European regulatory conundrum of phage therapy*. Future Microbiol* 2007, **2**:485-491.

**7) probiotic.mp. or probiotic agent AND informed consent**

**No references**

**8) probiotics.mp. or probiotic agent AND temperature/ or drug storage/ or drug packaging/ or cold chain.mp. or drug stability/ or freezing/**

1. Brinques GB, Do Carmo Peralba M, Ayub MAZ: Optimization of probiotic and lactic acid production by Lactobacillus plantarum in submerged bioreactor systems. *J Ind Microbiol* *Biotechnol* 2010, **37**:205-212.

2. Li XY, Chen XG, Cha DS, Park HJ, Liu CS: Microencapsulation of a probiotic bacteria with alginategelatin and its properties. *J Microencapsulation* 2009, **26**:315-324.

3. Lopez-Rubio A, Sanchez E, Sanz Y, Lagaron JM: Encapsulation of living bifidobacteria in ultrathin PVOH electrospun fibers. *Biomacromolecules* 2009, **10**:2823-2829.

4. Zapata S, Munoz J, Ruiz OS, Montoya OI, Gutierez PA: Isolation of Lactobacillus plantarum LPBM10 and partial characterization of its bacteriocin. *Vitae* 2009, **16**:75-82.

5. Bora PS, Puri V, Bansal AK: Physicochemical properties and excipient compatibility studies of probiotic Bacillus coagulans spores. *Sci Pharm* 2009, **77**:625-637.

6. Comas-Riu J, Rius N: Flow cytometry applications in the food industry. J *Ind Microbiol Biotechnol* 2009, **36**:999-1011.

7. Klayraung S, Viernstein H, Okonogi S: Development of tablets containing probiotics: Effects of formulation and processing parameters on bacterial viability. *Int J Pharm* 2009, **370**:54-60.

8. Albertini B, Vitali B, Passerini N, Cruciani F, Di Sabatino M, Rodriguez L, Brigidi P: Development of microparticulate systems for intestinal delivery of Lactobacillus acidophilus and Bifidobacterium lactis. *Eur J Pharm Sci* 2010, **40**:359-366.

9. Hebrard G, Hoffart V, Beyssac E, Cardot JM, Alric M, Subirade M: Coated whey protein/alginate microparticles as oral controlled delivery systems for probiotic yeast. *J Microencapsulation* 2010, **27**:292-302.

10. Kasra-Kermanshahi R, Fooladi J, Peymanfar S: Isolation and microencapsulation of Lactobacillus spp. from corn silage for probiotic application. *Iran J Microbiol* 2010, **2**:98-102.

11. Komatsu TR, Buriti FCA, Saad SMI: Overcoming hurdles through innovation, persistence and creativeness in the development of probiotic foods. (In Portuguese) *Rev Bras Cienc Farm* 2008, **44**:329-347.

12. Bansal T, Garg S. Probiotics: From functional foods to pharmaceutical products. *Curr Phar Biotechnol* 2008, **9**:267-287.

13. Sutton A: Product development of probiotics as biological drugs. *Clin Infect Di* 2008, **46**(Suppl 2):S128-S132.

14. Ding WK, Shah NP: Acid, bile, and heat tolerance of free and microencapsulated probiotic bacteria. *J Food Sci* 2007, **72**:M446-M450.

15. Reid AA, Champagne CP, Gardner N, Fustier P, Vuillemard JC: Survival in food systems of Lactobacillus rhamnosus R011 microentrapped in whey protein gel particles. *J Food Sci* 2007, **72**:M031-037.

16. Oliveira AC, Moretti TS, Boschini C, Baliero JC, Freitas O, Favaro-Trindade CS: Stability of microencapsulated B. lactis (BI 01) and L. acidophilus (LAC 4) by complex coacervation followed by spray drying. J Microencapsul 2007, **24**:673-681.

17. Weissbrodt J, Kunz B: Influence of hydrocolloid interactions on their encapsulation properties using spray-drying. *Minerva Biotecnol* 2007, **19**:27-32.

18. Collado MC, Sanz Y: Induction of acid resistance in Bifidobacterium: A mechanism for improving desirable traits of potentially probiotic strains. *J Appl Microbiol* 2007, **103**:1147-1157.

19. Otieno DO, Shah NP: Endogenous beta-glucosidase and beta-galactosidase activities from selected probiotic micro-organisms and their role in isoflavone biotransformation in soymilk*. J Appl Microbiol* 2007, **103**:910-917.

20. Oliveira AC, Moretti TS, Boschini C, Baliero JC, Freitas O, Favaro-Trindade CS: Stability of microencapsulated B. lactis (BI 01) and L. acidophilus (LAC 4) by complex coacervation followed by spray drying. *J Microencapsulation* 2007, **24**:685-693.

21. Mortazavian AM, Ehsani MR, Mousavi SM, Rezaei K, Sohrabvandi S, Reinheimer JA: Effect of refrigerated storage temperature on the viability of probiotic micro-organisms in yogurt. *Int J Dairy Technol* 2007, **60**:123-127.

22. Annan NT, Borza A, Moreau DL, Allan-Wojtas PM, Hansen LT: Effect of process variables on particle size and viability of Bifidobacterium lactis Bb-12 in genipin-gelatin microspheres. *J Microencapsulation* 2007, **24**:152-162.

23. Dongowski G, Jacobasch G, Schmiedl D: Structural stability and prebiotic properties of resistant starch type 3 increase bile acid turnover and lower secondary bile acid formation. *J Agric Food Chem* 2005, **53**:9257-9267.

24. Korakoch H, Prakit S, Gerard L, Sunee N, Didier M, Penkhae W: Prediction on the stability of spray-dried Lactobacillus reuteri KUB-AC5 by Arrhenius equation for long-term storage. *J Microbiol Biotechnol* 2005, **15**:1178-1182.

25. Drisko J, Bischoff B, Giles C, Adelson M, Rao RV, McCallum R: Evaluation of five probiotic products for label claims by DNA extraction and polymerase chain reaction analysis. *Dig Dis Sci* 2005, **50**:1113-1117.

26. Wang Y, Delettre J, Guillot A, Corrieu G, Béal C: Influence of cooling temperature and duration on cold adaptation of Lactobacillus acidophilus RD758. *Cryobiology* 2005, **50**:294-307.

27. Talwalkar A, Kailasapathy K: The role of oxygen in the viability of probiotic bacteria with reference to L. acidophilus and Bifidobacterium spp. *Curr Issues Intest Microbiol* 2004, **5**:1-8.

28. Helland MH, Wicklund T, Narvhus JA: Growth and metabolism of selected strains of probiotic bacteria, in maize porridge with added malted barley. *Int J Food Microbiol* 2004, **91**:305-313.

29. Prasad J, McJarrow P, Gopal P: Heat and osmotic stress responses of probiotic Lactobacillus rhamnosus HN001 (DR20) in relation to viability after drying. *Appl Environ Microbiol* 2003, **69**:917-925.

30. Favaro-Trinidade CS, Grosso CR: Microencapsulation of L. acidophilus (La-05) and B. lactis (Bb-12) and evaluation of their survival at the pH values of the stomach and in bile. *J Microencapsulation* 2002, **19**:485-494.

31. Nebra Y, Jofre J, Blanch AR: The effect of reducing agents on the recovery of injured Bifidobacterium cells. *J Microbiol Methods* 2002, **49**:247-254.

32. Adhikari K, Mustapha A, Grun IU, Fernando L: Viability of microencapsulated bifidobacteria in set yogurt during refrigerated storage. *J Dairy Sci* 2000, **83**:1946-1951.

33. Davidson RH, Duncan SE, Hackney CR, Eigel WN, Boling JW: Probiotic culture survival and implications in fermented frozen yogurt characteristics. *J Dairy Sci* 2000, **83**:666-673.

34. Cui JH, Goh JS, Kim PH, Choi SH, Lee BJ: Survival and stability of bifidobacteria loaded in alginate poly-l-lysine microparticles. *Int J Pharm* 2000, **210**:51-59.

35. Elofsson U, Millqvist-Fureby A: Drying of probiotic micro-organisms in aqueous two-phase systems. *Minerva Biotechnol* 2000, **12**:279-286.

36. Sultana K, Godward G, Reynolds N, Arumugaswamy R, Peiris P, Kailasapathy K: Encapsulation of probiotic bacteria with alginate-starch and evaluation of survival in simulated gastrointestinal conditions and in yoghurt. *Int J Food Microbiol* 2000, **62**:47-55.

37. Klein G, Pack A, Bonaparte C, Reuter G: Taxonomy and physiology of probiotic lactic acid bacteria. *Int J Food Microbiol* 1998, **41**:103-125.

**9) probiotic.mp. or probiotic agent quality assurance.mp. or quality control/**

1. Vanhee LME, Goeme F, Nelis HJ, Coenye T: [Quality control of fifteen probiotic products containing Saccharomyces boulardii.](http://ovidsp.tx.ovid.com.pklibresources.health.wa.gov.au/sp-3.4.1a/ovidweb.cgi?&S=JMIDFPJJCADDMCIGNCCLAHJCMDMPAA00&Complete+Reference=S.sh.20|6|1) *Journal of Applied Microbiology* 2010, **109**:1745-1752.

2. Elliott E, Teversham K: [An evaluation of nine probiotics available in South Africa, August 2003.](http://ovidsp.tx.ovid.com.pklibresources.health.wa.gov.au/sp-3.4.1a/ovidweb.cgi?&S=JMIDFPJJCADDMCIGNCCLAHJCMDMPAA00&Complete+Reference=S.sh.20|54|1) *South African Medical Journal* 2004, **94**:121-124.

3. Sanz Y., Collado M.C., Dalmau J. [Probiotics: Quality criteria and guidelines for their use.](http://ovidsp.tx.ovid.com.pklibresources.health.wa.gov.au/sp-3.4.1a/ovidweb.cgi?&S=JMIDFPJJCADDMCIGNCCLAHJCMDMPAA00&Complete+Reference=S.sh.20|59|1) (Probioticos: Criterios de calidad y orientaciones para el consumo.) *Acta Pediatrica Espanola* 2003, **61**:476-482.

4. Szajewska H, Fordymacka A, Banaszkiewicz A. [Microbiological qualitative and quantitative analysis of probiotic products registered in Poland for medicinal purposes (pilot study).](http://ovidsp.tx.ovid.com.pklibresources.health.wa.gov.au/sp-3.4.1a/ovidweb.cgi?&S=JMIDFPJJCADDMCIGNCCLAHJCMDMPAA00&Complete+Reference=S.sh.20|65|1) (Ocena *mikrobiologiczna preparatow probiotycznych zarejestrowanych w polsce (badanie wstepne).) Pediatria* Polska 2002, **77**:941-944.
